# Supplementary figures and images for: ACVR2A facilitates trophoblast cell invasion through TCF7/c-JUN pathway in pre-eclampsia progression
Source: eLife. 2025 May 30;14:RP101236. doi: 10.7554/eLife.101236 (PMC12124833; doi:10.7554/eLife.101236)

GAPDH

NC

PE

36KDa

内参 重组

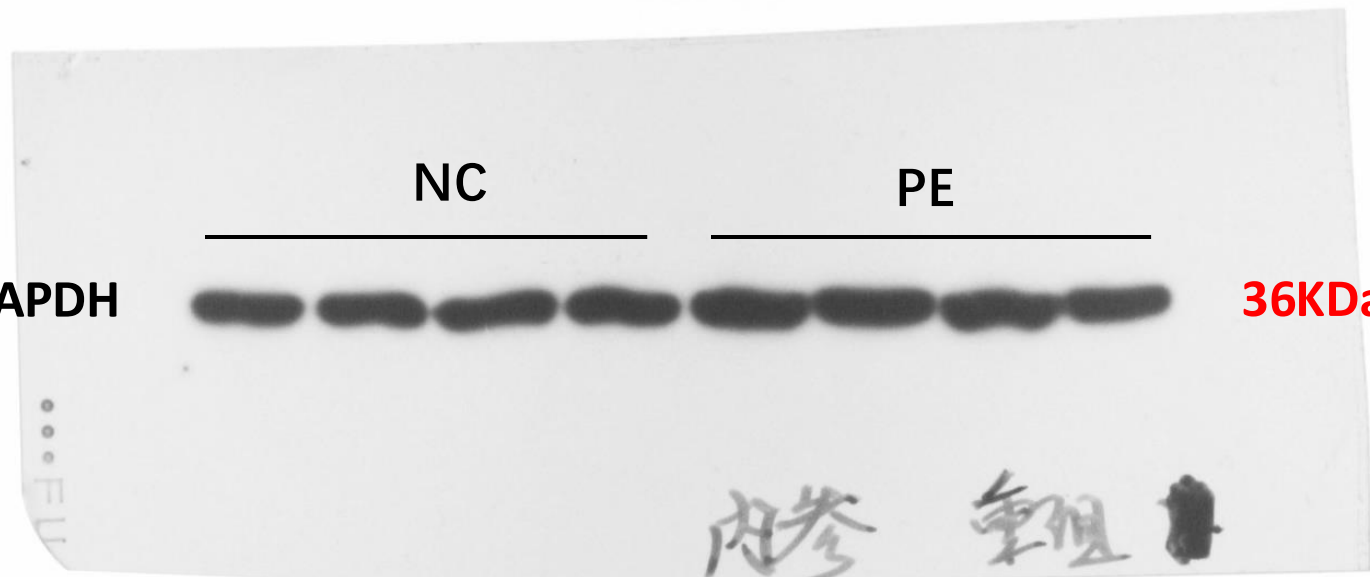

Supplement: Figure 1—source data 1. [file elife-101236-fig1-data1.zip › Figure 1C-source data 1.pdf]

ACVR2A

NC

PE

58KDa

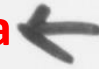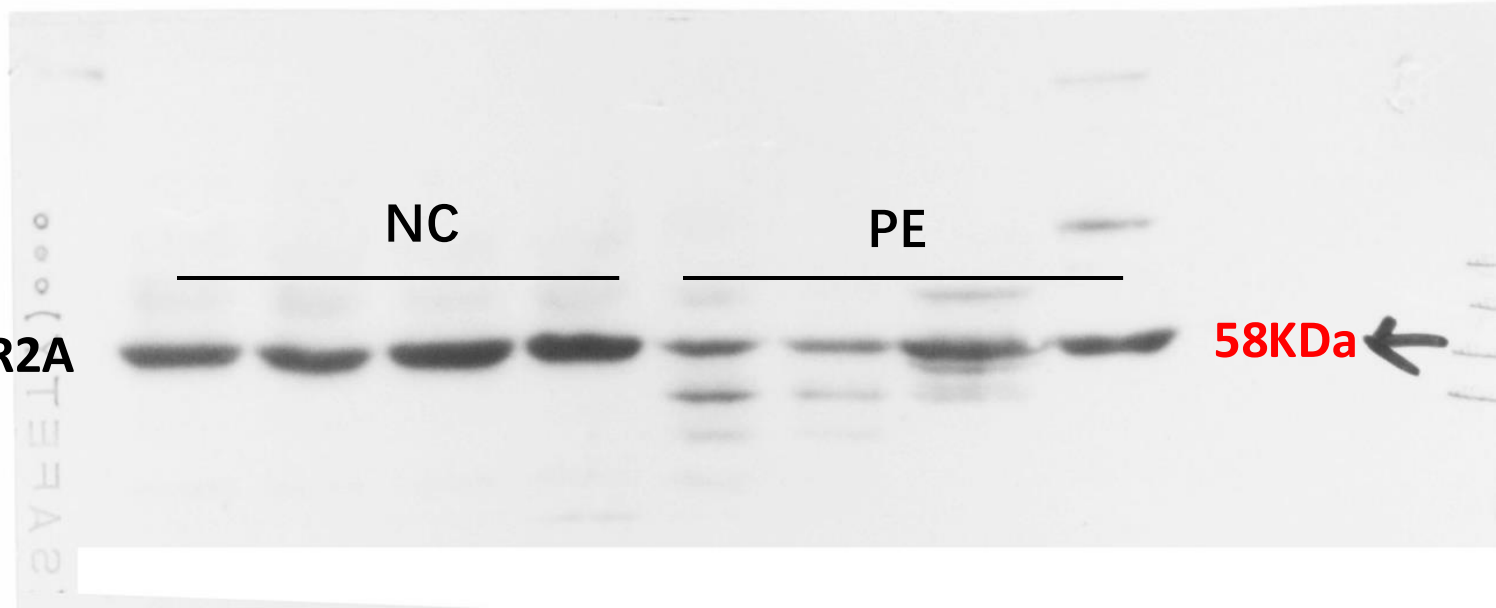

Supplement: Figure 1—source data 1. [file elife-101236-fig1-data1.zip › Figure 1C-source data 2.pdf]

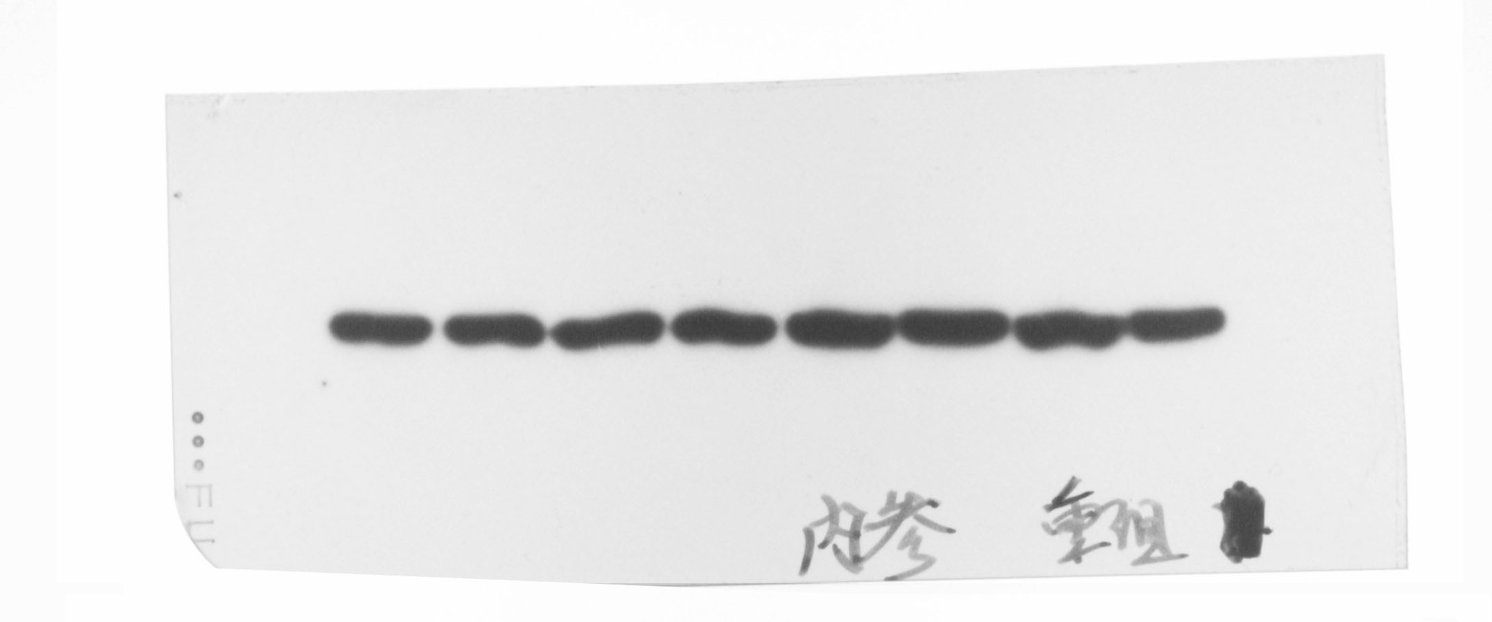

Supplement: Figure 1—source data 2. [file elife-101236-fig1-data2.zip › Figure 1C-source data 1.pic.jpg]

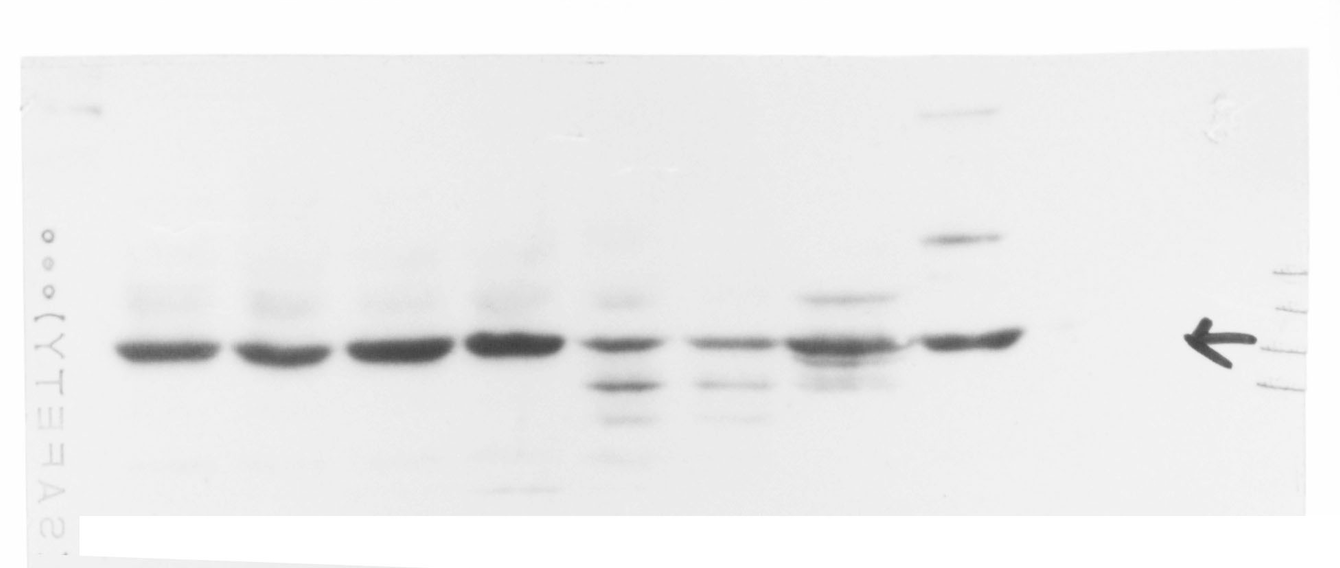

Supplement: Figure 1—source data 2. [file elife-101236-fig1-data2.zip › Figure 1C-source data 2.pic.jpg]

130  
95  
70  
55  
43  
33

第-2组 ACVR2A

=3组 ACVR2A

6  
5  
5  
5

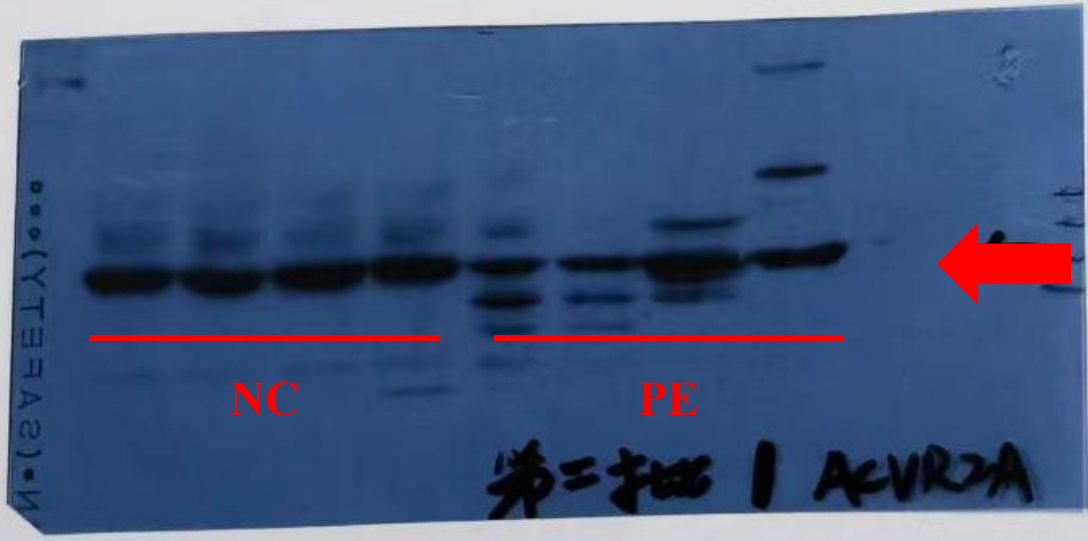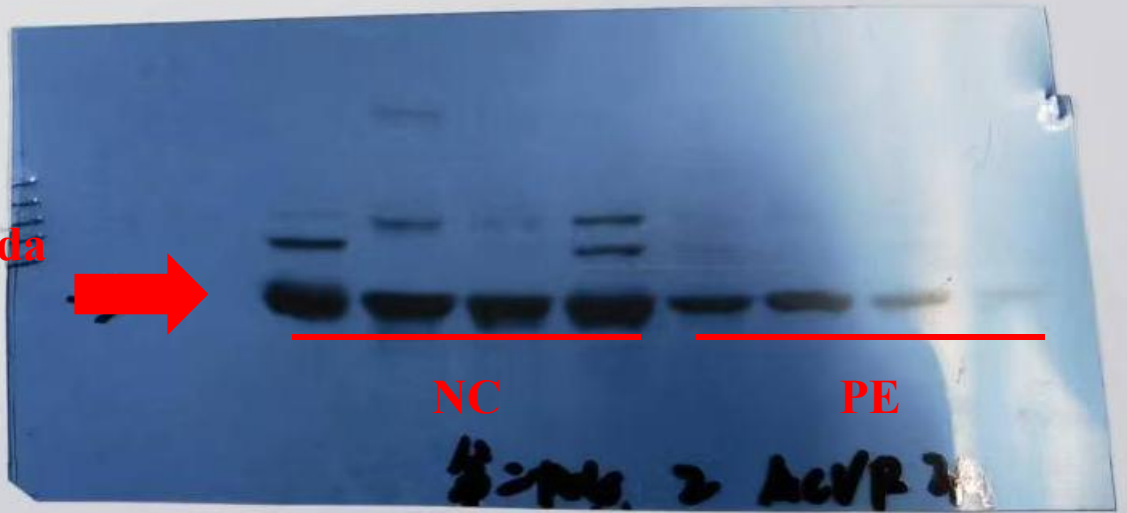

Supplement: Figure 1—figure supplement 1—source data 1. [file elife-101236-fig1-figsupp1-data1.pdf]

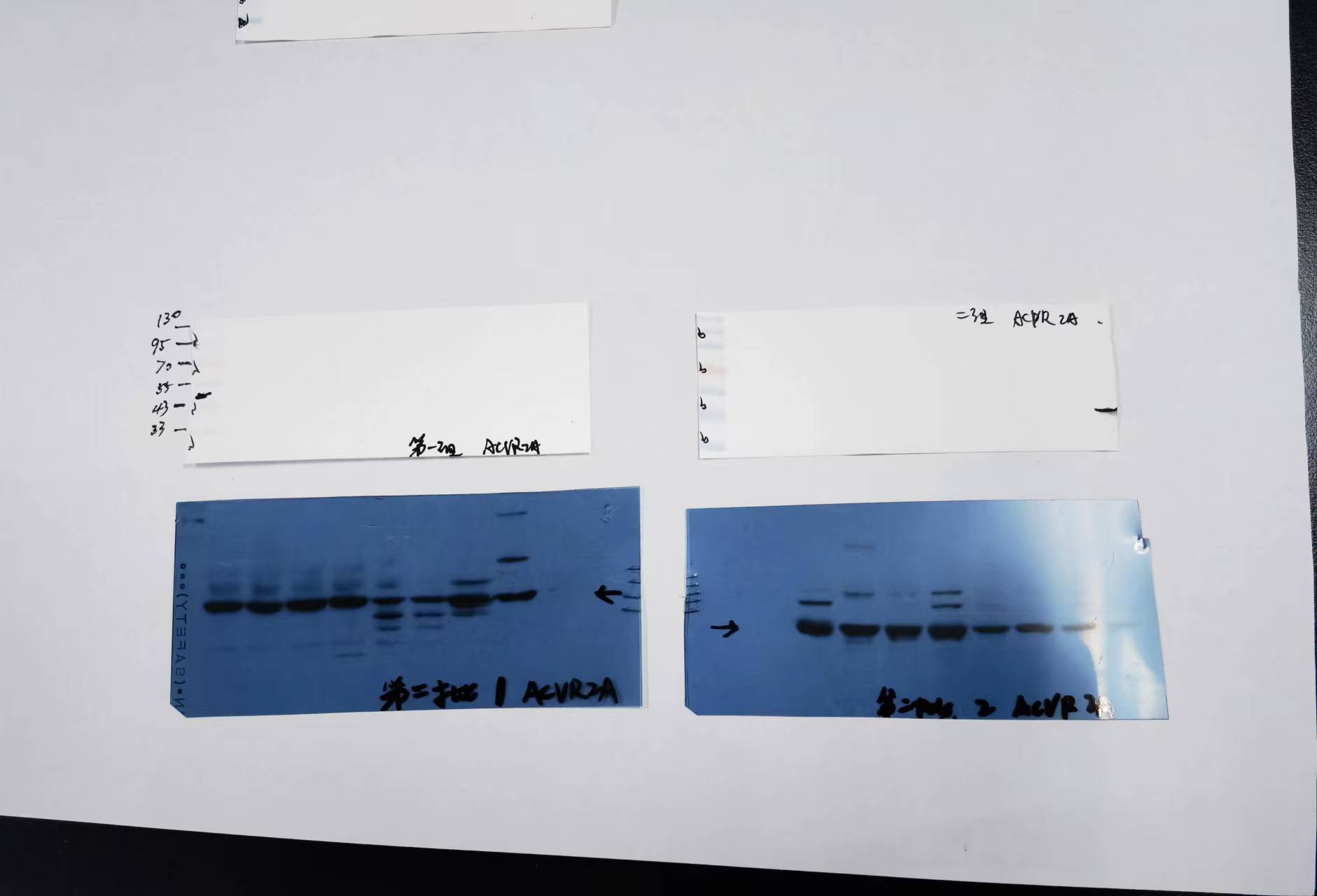

Supplement: Figure 1—figure supplement 1—source data 2. [file elife-101236-fig1-figsupp1-data2.zip › Figure 1 - Figure supplement 1-source data 1.jpg]

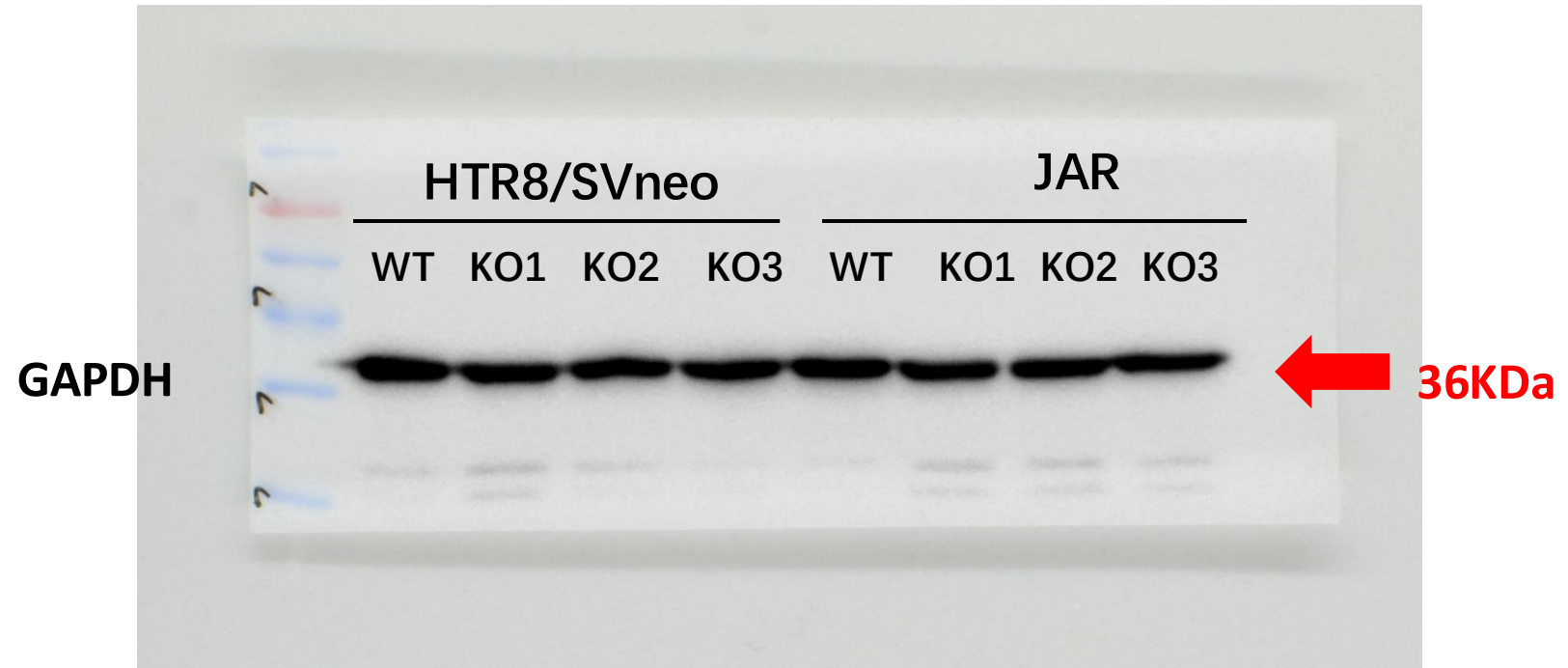

Supplement: Figure 2—source data 1. [file elife-101236-fig2-data1.zip › Figure 2E-source data 1.pdf]

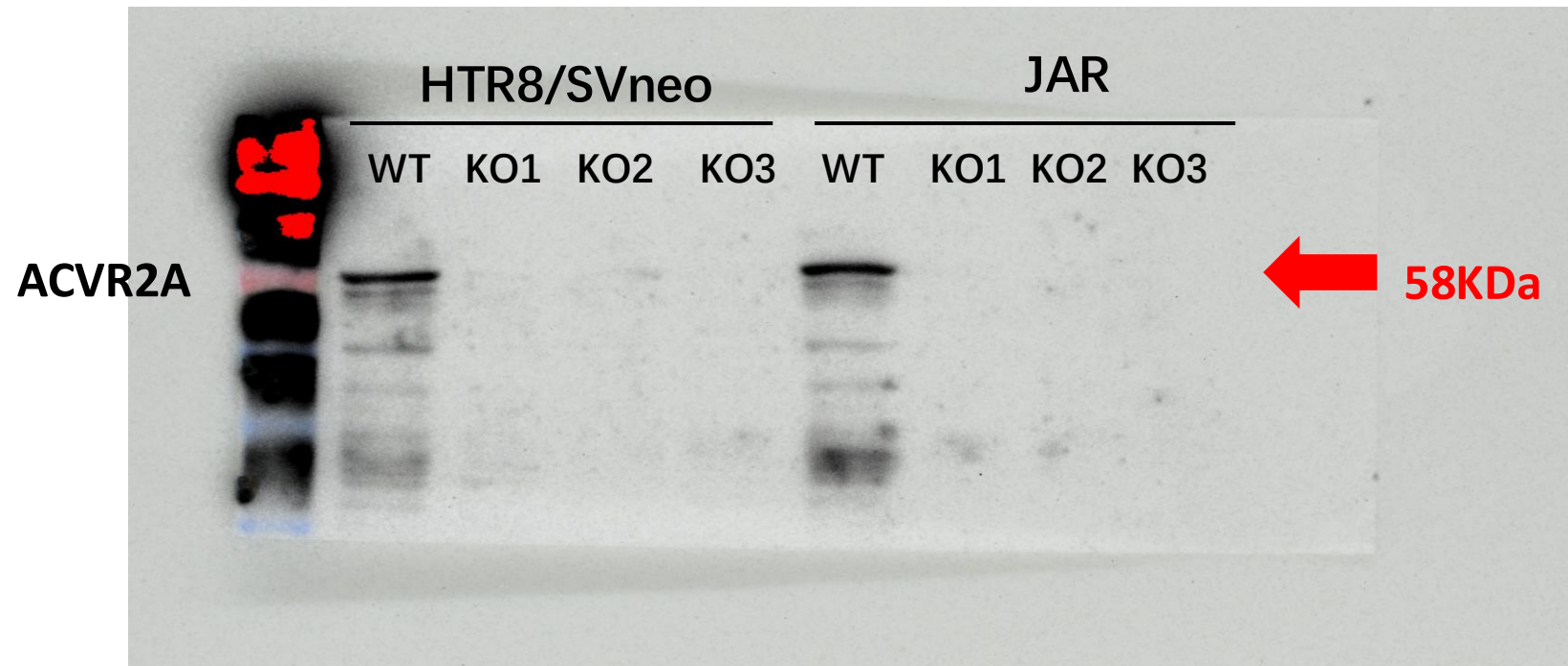

Supplement: Figure 2—source data 1. [file elife-101236-fig2-data1.zip › Figure 2E-source data 2.pdf]

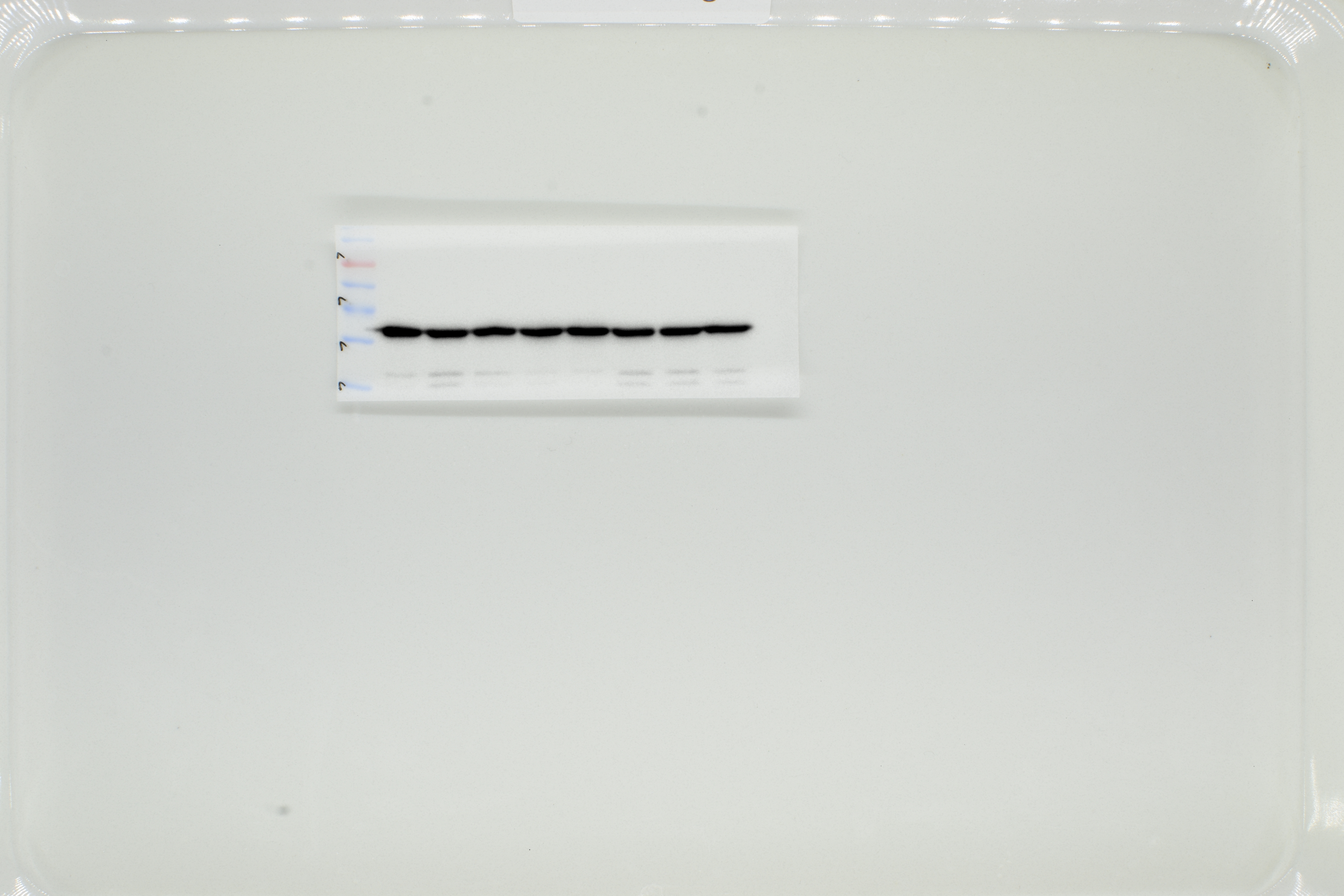

Supplement: Figure 2—source data 2. [file elife-101236-fig2-data2.zip › Figure 2E-source data 1.jpg]

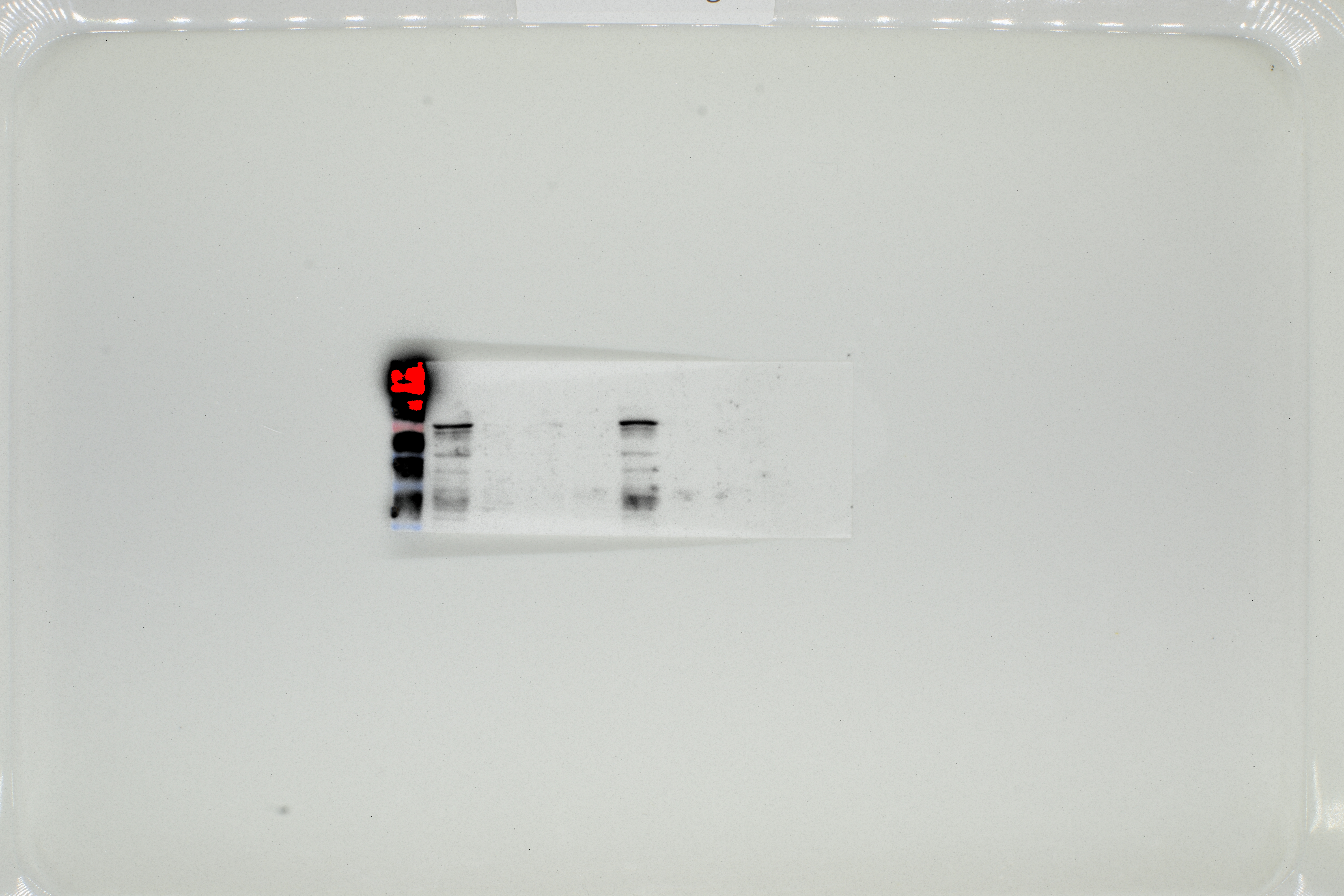

Supplement: Figure 2—source data 2. [file elife-101236-fig2-data2.zip › Figure 2E-source data 2.jpg]

**Figure 2 - Figure supplement 3 source data 1 A**

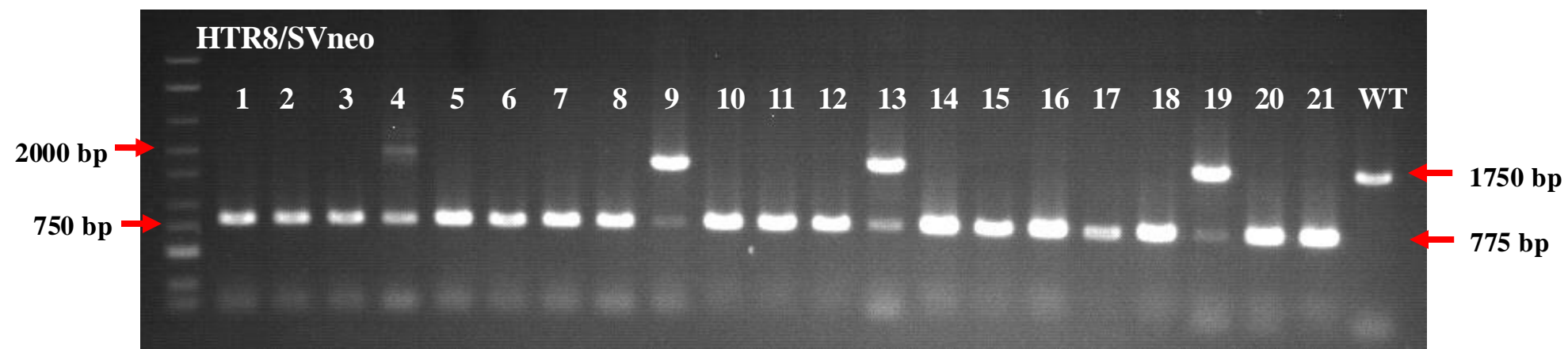

Supplement: Figure 2—figure supplement 1—source data 1. [file elife-101236-fig2-figsupp1-data1.zip › Figure 2 - Figure supplement 3 source data 1.pdf]

Figure 2 - Figure supplement 3 source data 1 B

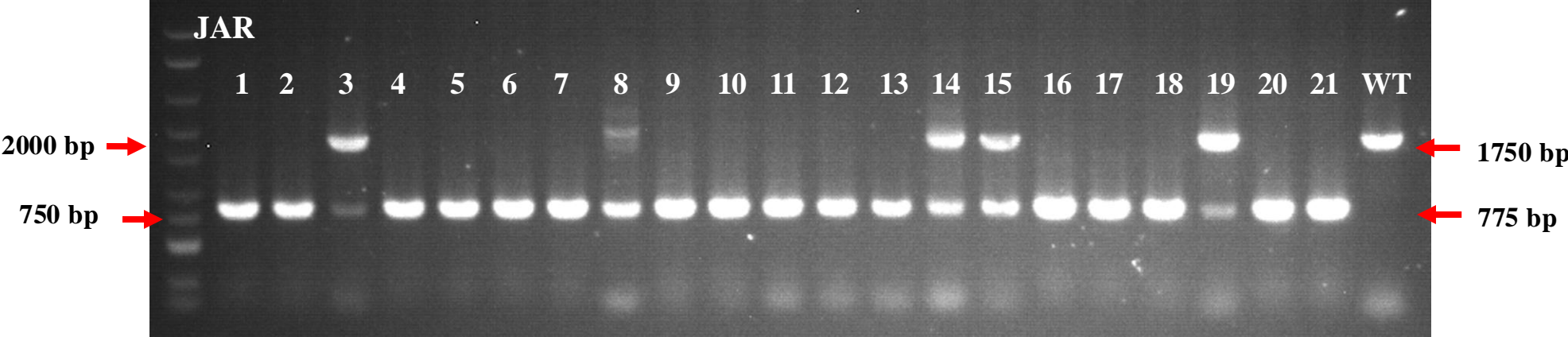

Supplement: Figure 2—figure supplement 1—source data 1. [file elife-101236-fig2-figsupp1-data1.zip › Figure 2 - Figure supplement 3 source data 2.pdf]

Figure 2 - Figure supplement 3 source data 1 C

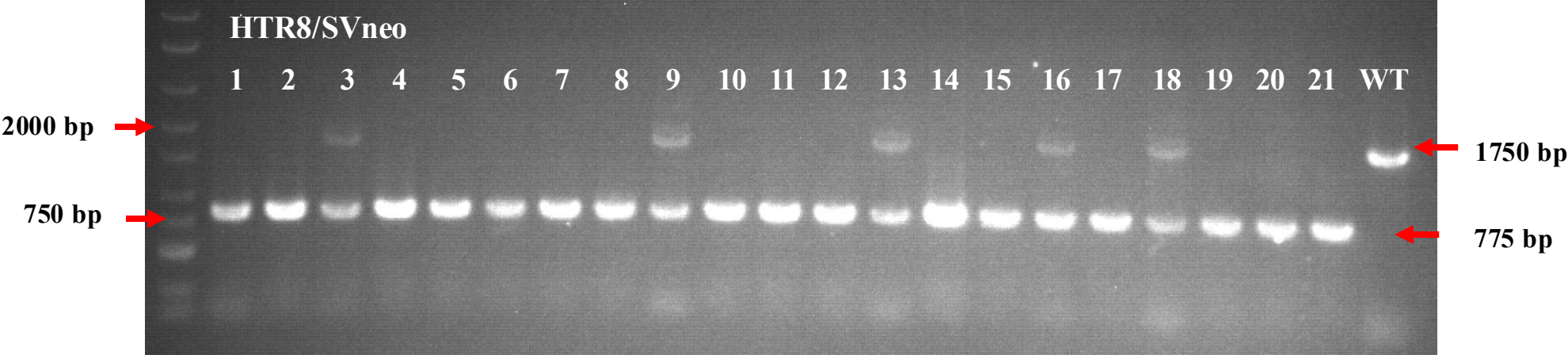

Supplement: Figure 2—figure supplement 1—source data 1. [file elife-101236-fig2-figsupp1-data1.zip › Figure 2 - Figure supplement 3 source data 3.pdf]

Figure 2 - Figure supplement 3 source data 1 D

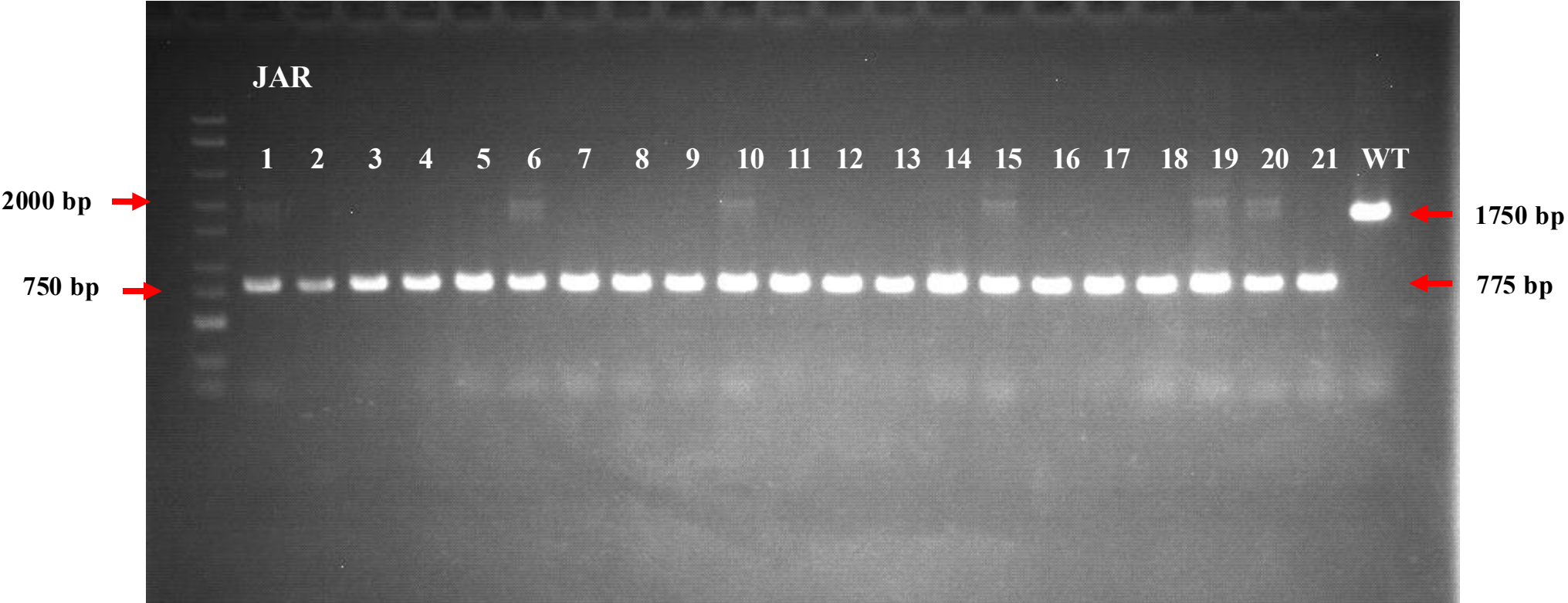

Supplement: Figure 2—figure supplement 1—source data 1. [file elife-101236-fig2-figsupp1-data1.zip › Figure 2 - Figure supplement 3 source data 4.pdf]

**Figure 2 - Figure supplement 3 source data 1 E and F**

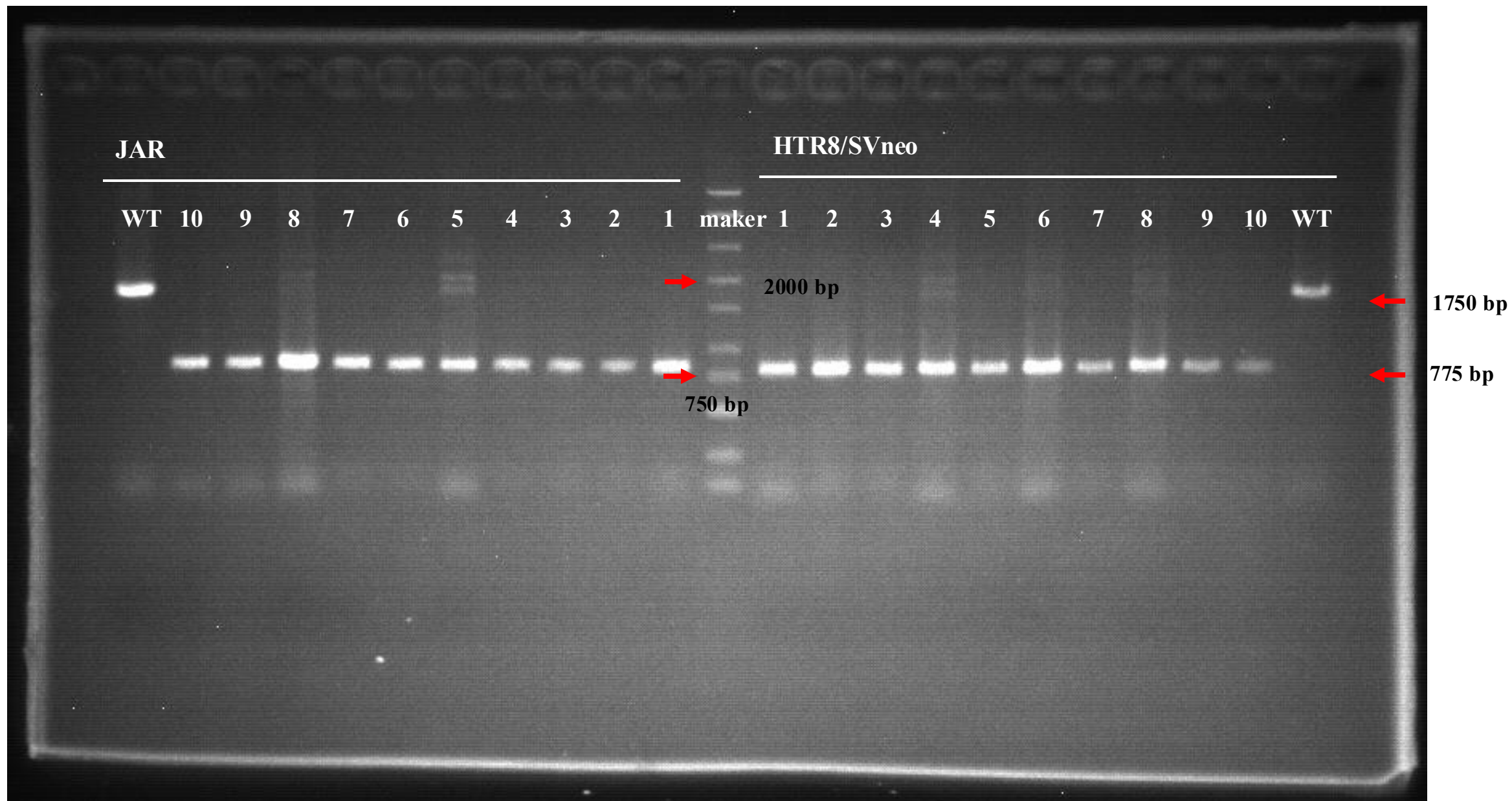

Supplement: Figure 2—figure supplement 1—source data 1. [file elife-101236-fig2-figsupp1-data1.zip › Figure 2 - Figure supplement 3 source data 5.pdf]

Figure 2 - Figure supplement 3 source data 1 G and H

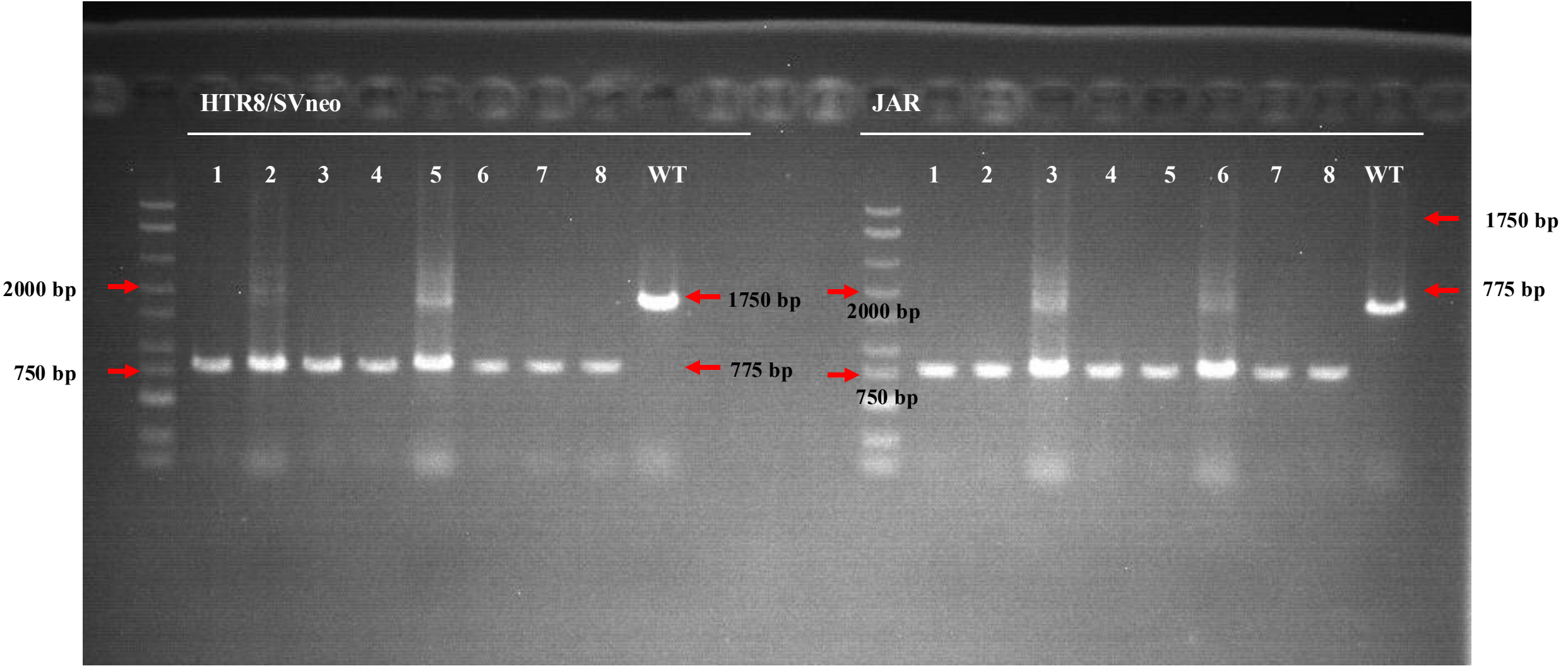

Supplement: Figure 2—figure supplement 1—source data 1. [file elife-101236-fig2-figsupp1-data1.zip › Figure 2 - Figure supplement 3 source data 6.pdf]

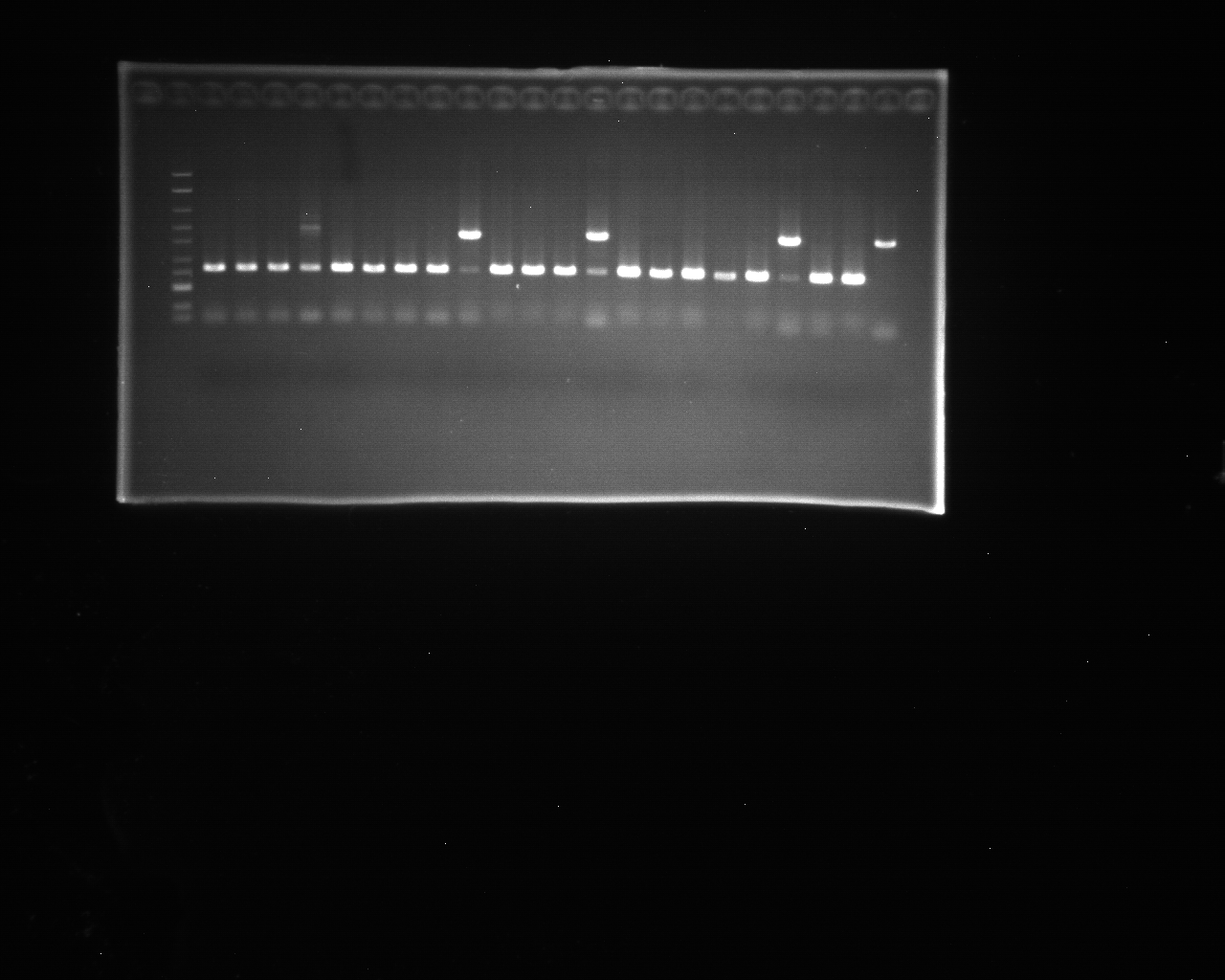

Supplement: Figure 2—figure supplement 1—source data 2. [file elife-101236-fig2-figsupp1-data2.zip › Figure 2 - Figure supplement 3 source data 1.Tif]

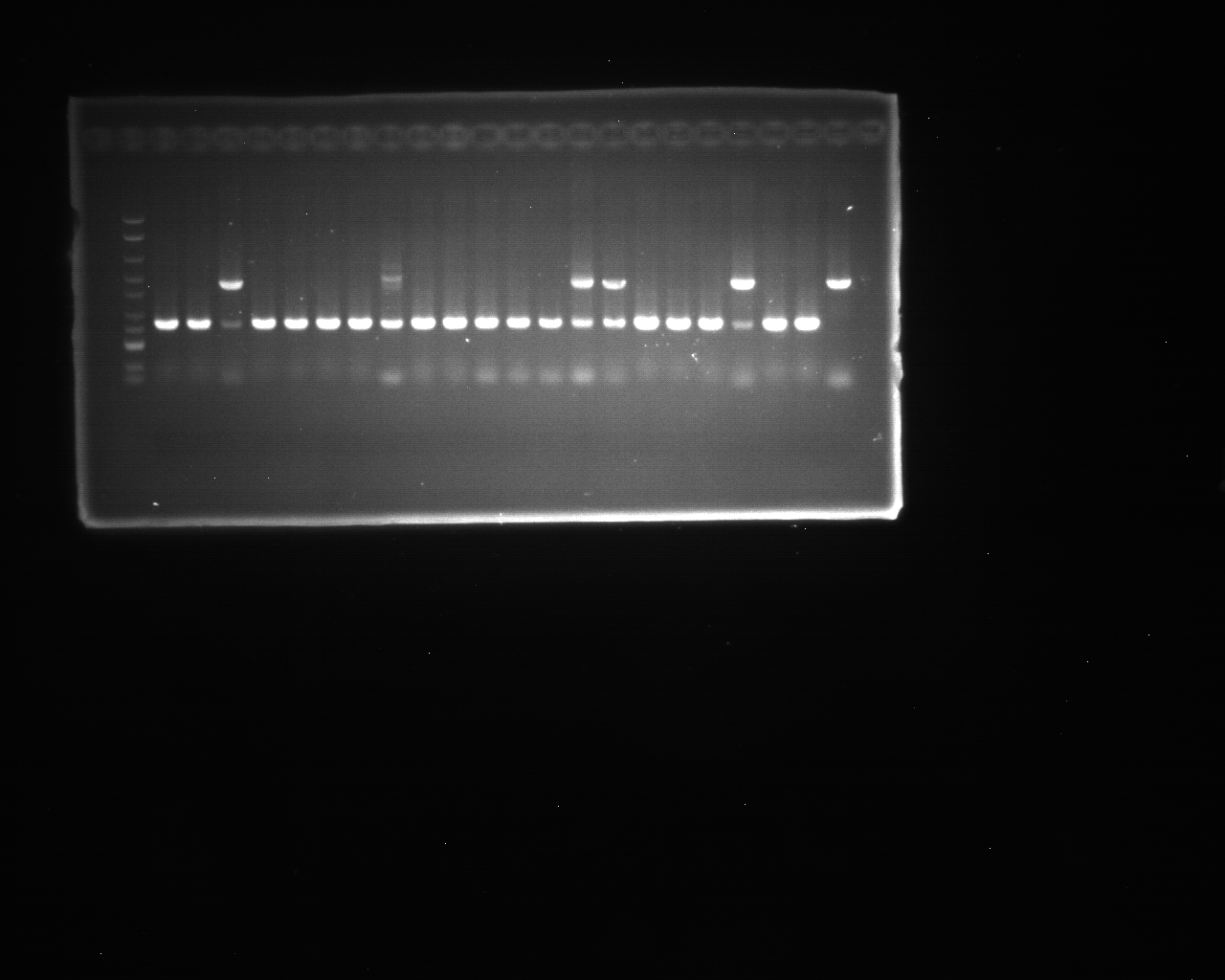

Supplement: Figure 2—figure supplement 1—source data 2. [file elife-101236-fig2-figsupp1-data2.zip › Figure 2 - Figure supplement 3 source data 2.Tif]

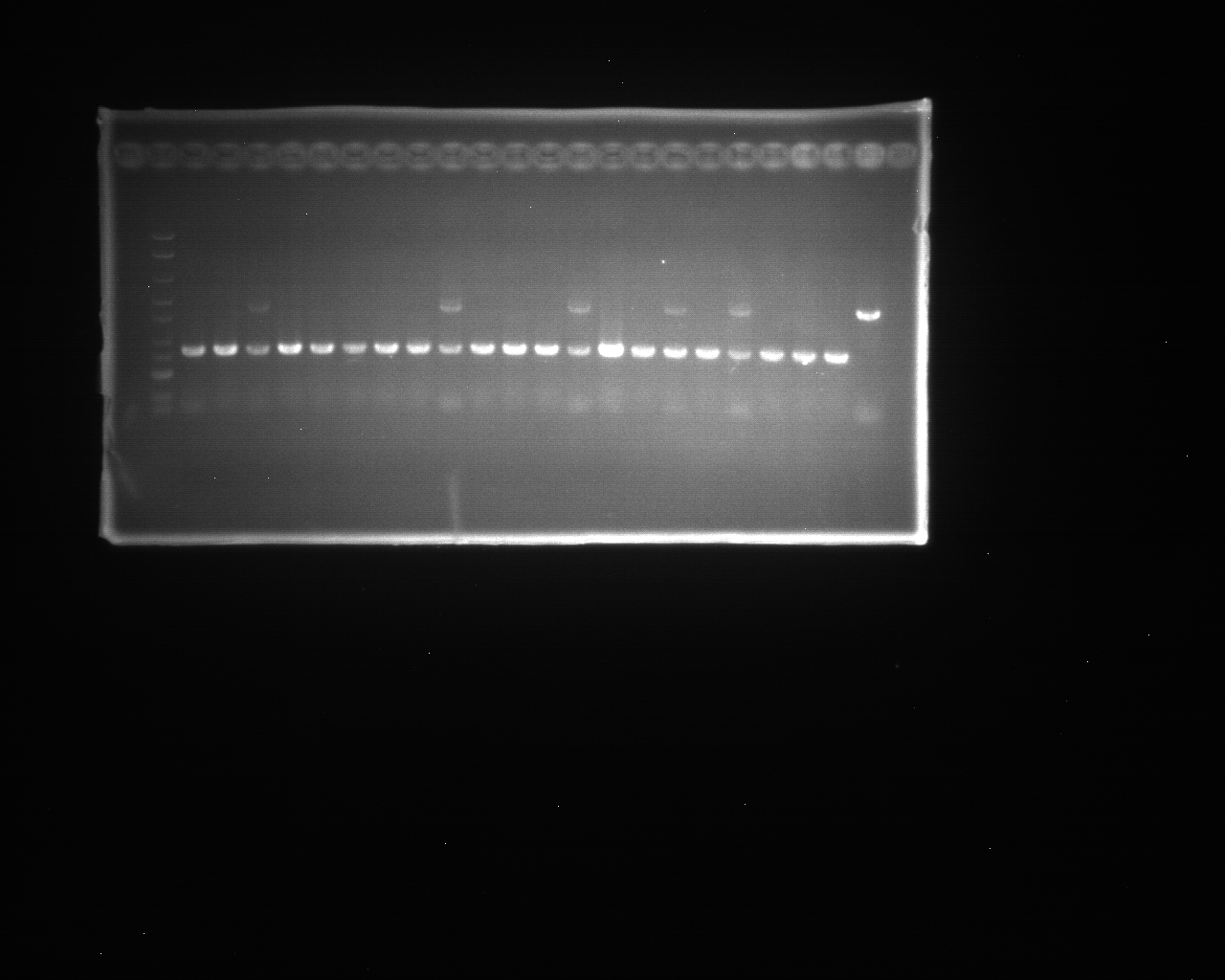

Supplement: Figure 2—figure supplement 1—source data 2. [file elife-101236-fig2-figsupp1-data2.zip › Figure 2 - Figure supplement 3 source data 3.Tif]

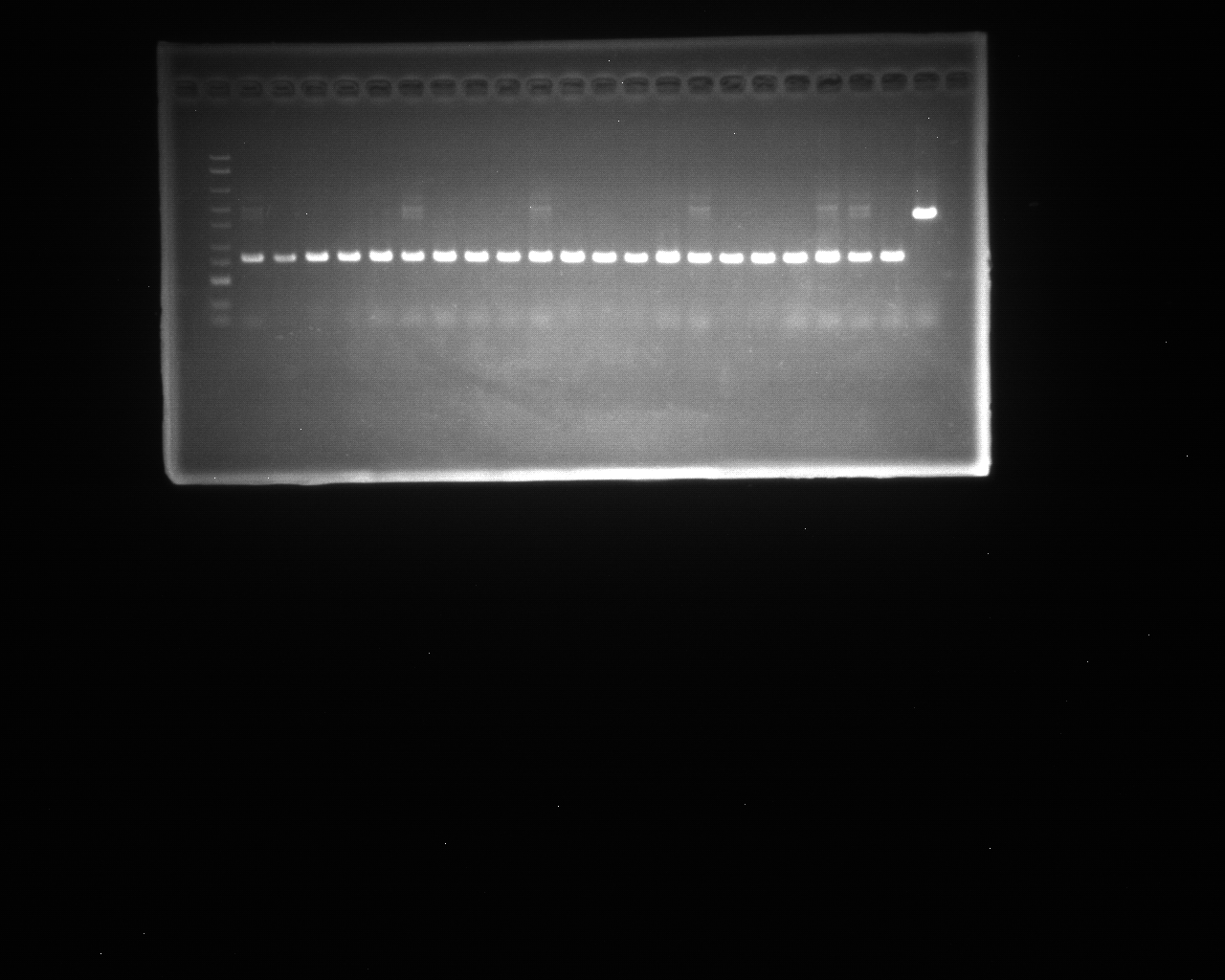

Supplement: Figure 2—figure supplement 1—source data 2. [file elife-101236-fig2-figsupp1-data2.zip › Figure 2 - Figure supplement 3 source data 4.Tif]

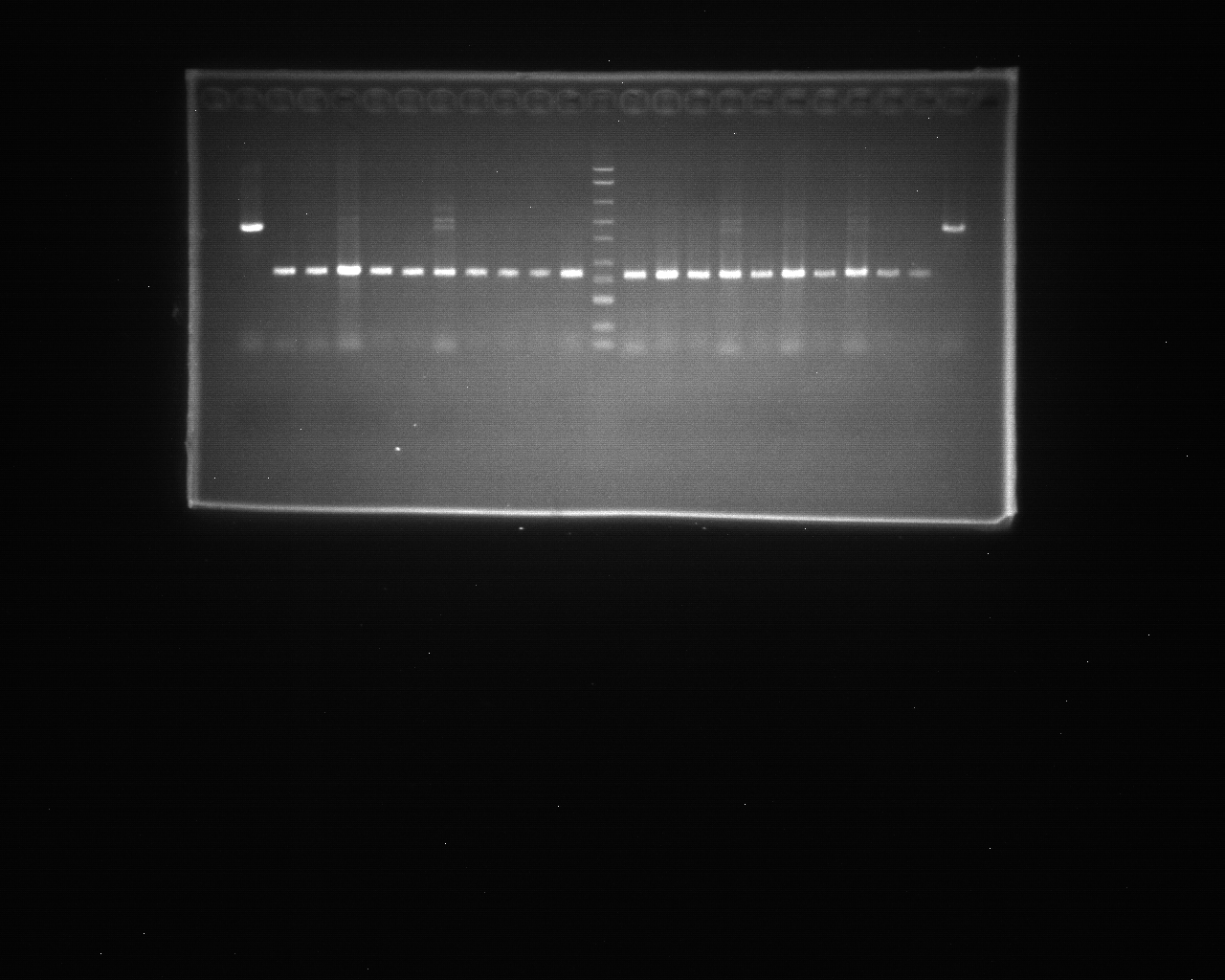

Supplement: Figure 2—figure supplement 1—source data 2. [file elife-101236-fig2-figsupp1-data2.zip › Figure 2 - Figure supplement 3 source data 5.Tif]

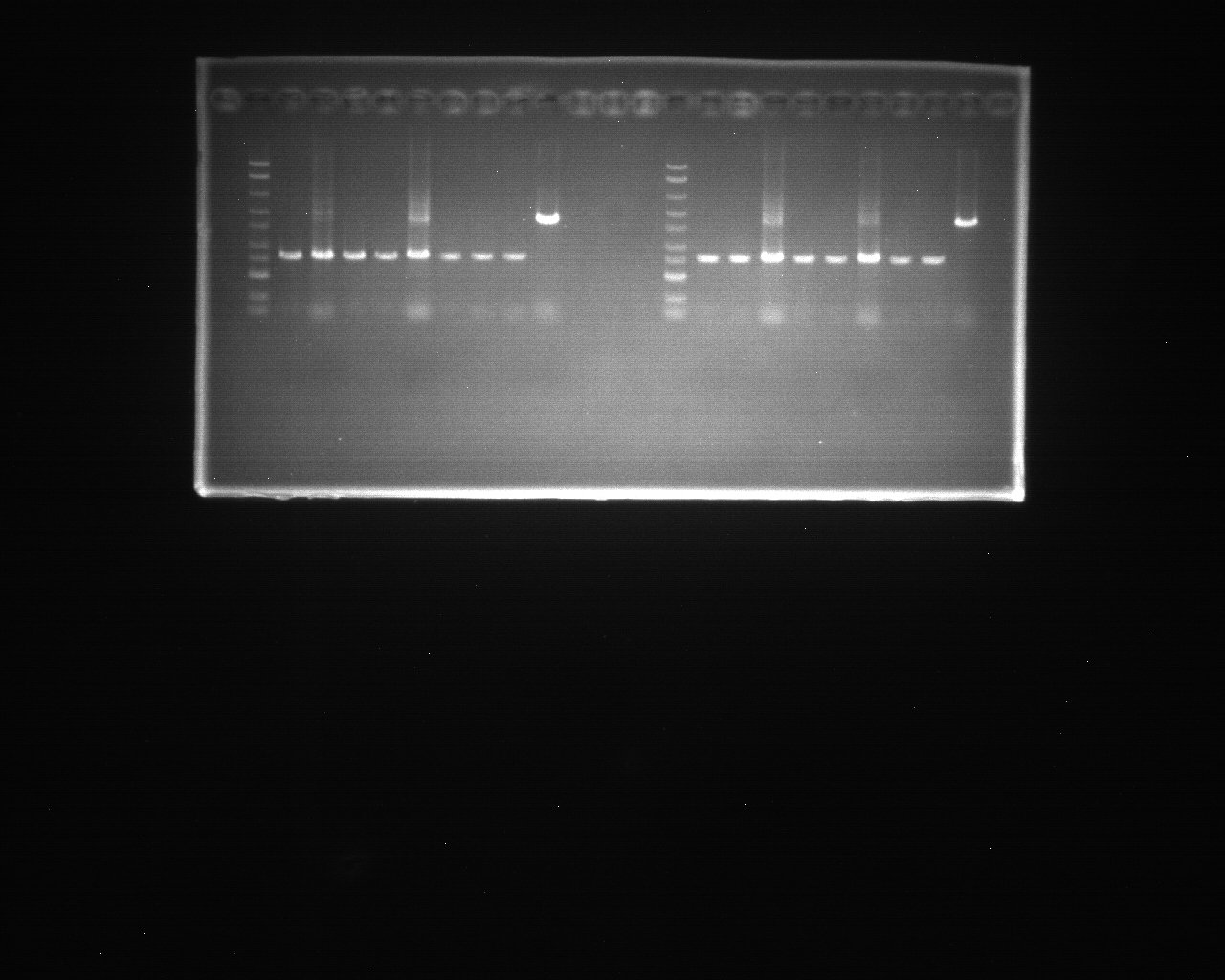

Supplement: Figure 2—figure supplement 1—source data 2. [file elife-101236-fig2-figsupp1-data2.zip › Figure 2 - Figure supplement 3 source data 6.Tif]

**Figure 7A**

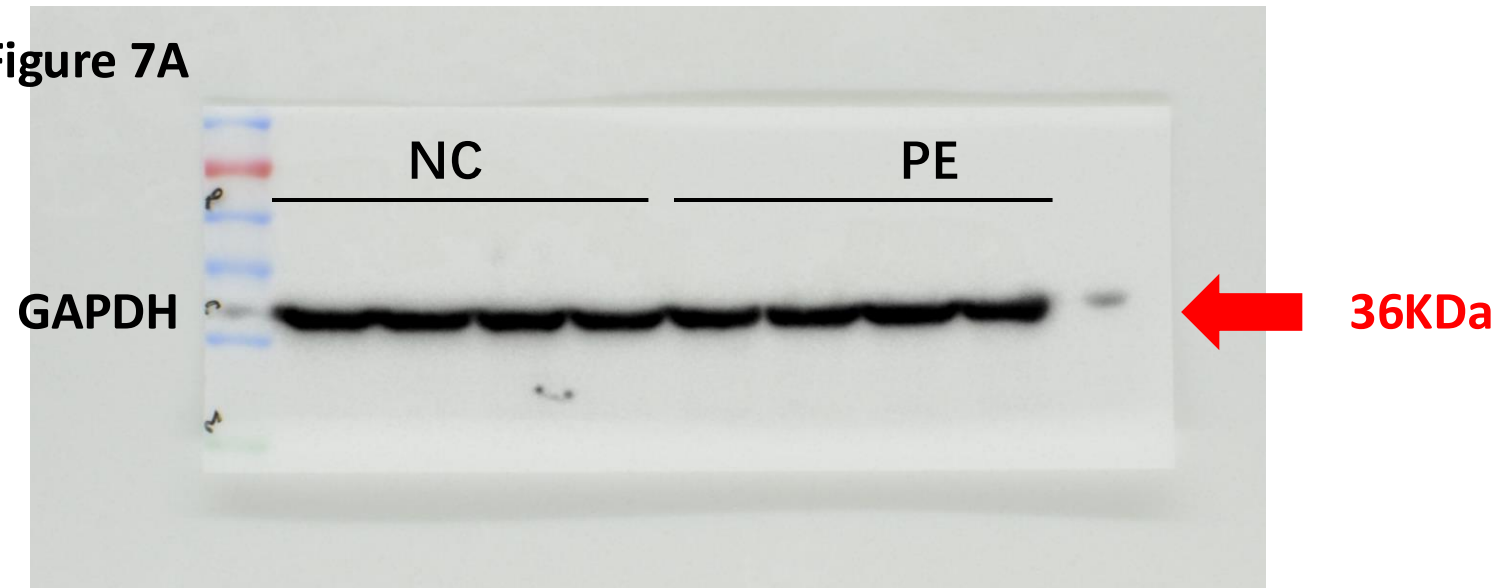

Supplement: Figure 7—source data 1. [file elife-101236-fig7-data1.zip › Figure 7A-ACVR2A source data 1.pdf]

Figure 7A

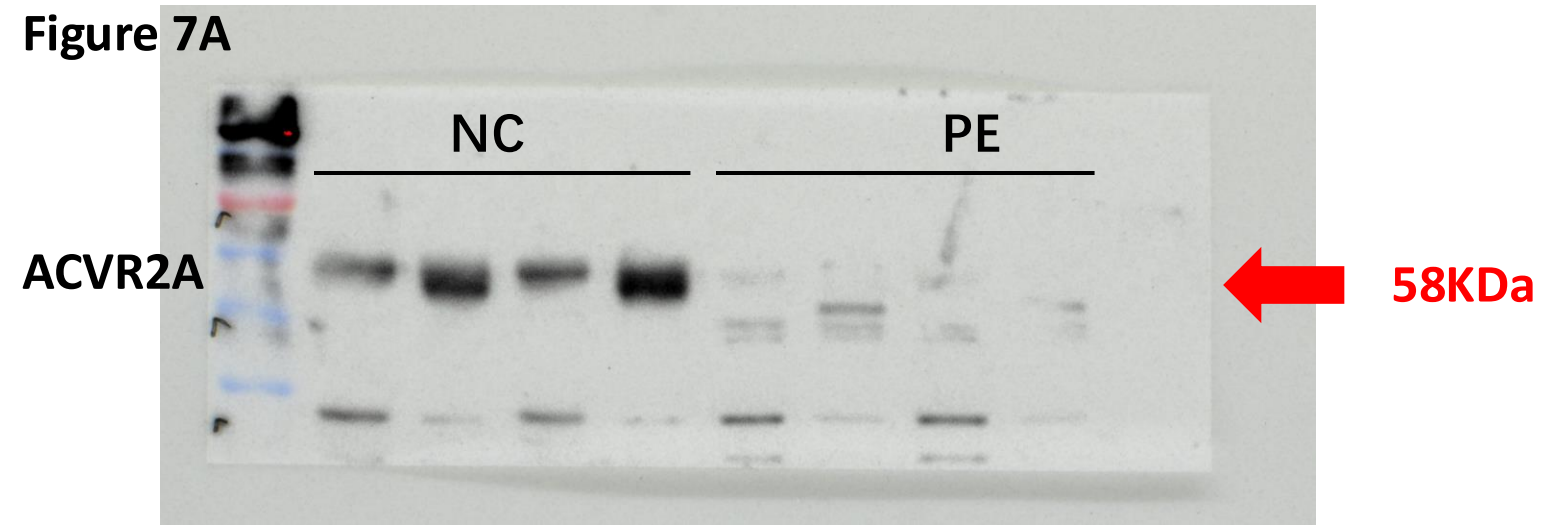

Supplement: Figure 7—source data 1. [file elife-101236-fig7-data1.zip › Figure 7A-ACVR2A source data 2.pdf]

**Figure 7A**

**pSMAD1/5/9**

**60KDa**

**NC**

**PE**

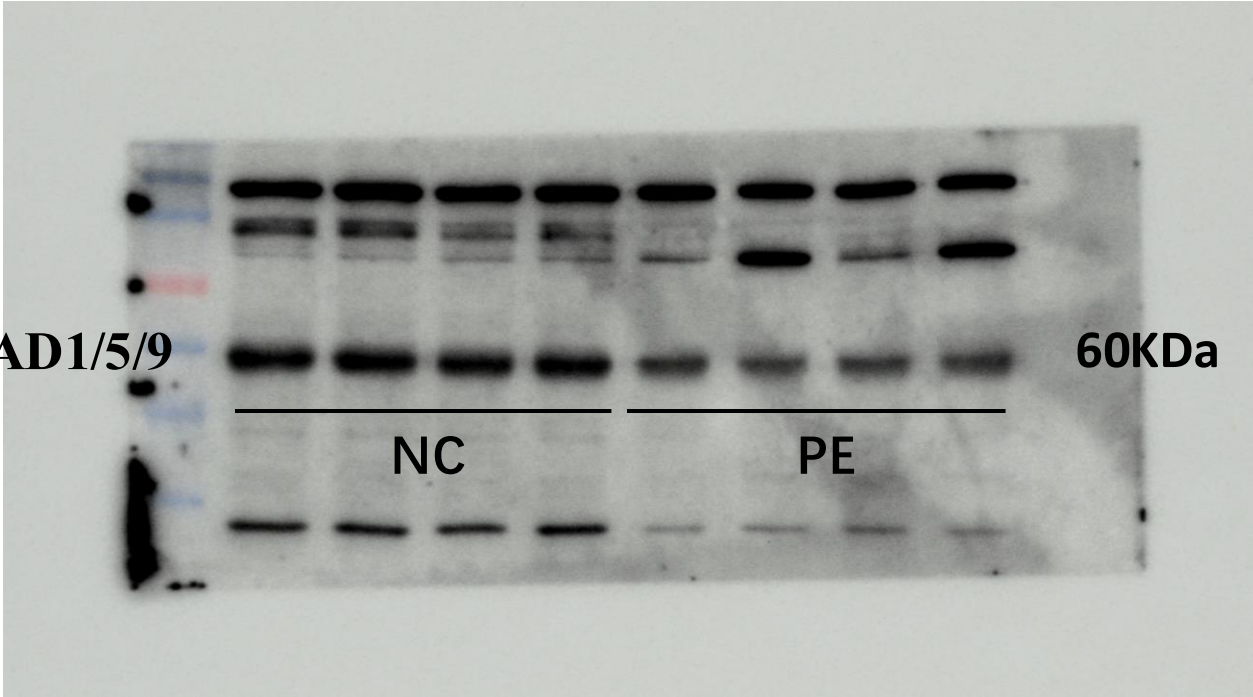

Supplement: Figure 7—source data 1. [file elife-101236-fig7-data1.zip › Figure 7A-pSMAD159 source data 1.pdf]

**Figure 7A**

**GAPDH**

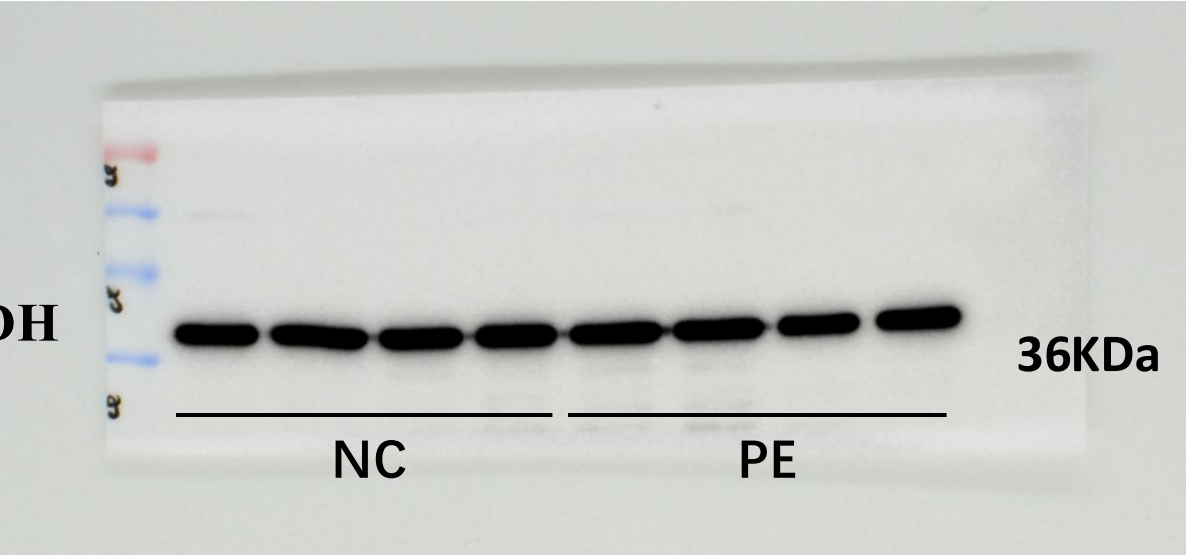

**NC**

**PE**

**36KDa**

Supplement: Figure 7—source data 1. [file elife-101236-fig7-data1.zip › Figure 7A-pSMAD159 source data 2.pdf]

**Figure 7A**

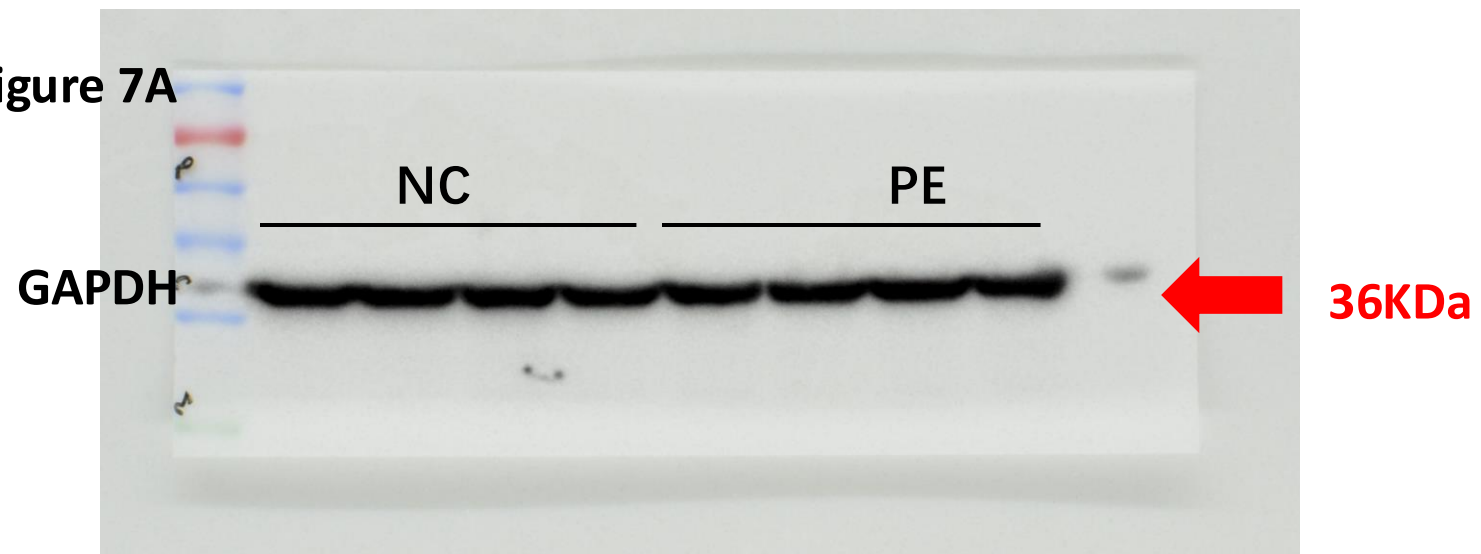

Supplement: Figure 7—source data 1. [file elife-101236-fig7-data1.zip › Figure 7A-SMAD4 source data 1.pdf]

Figure 7A

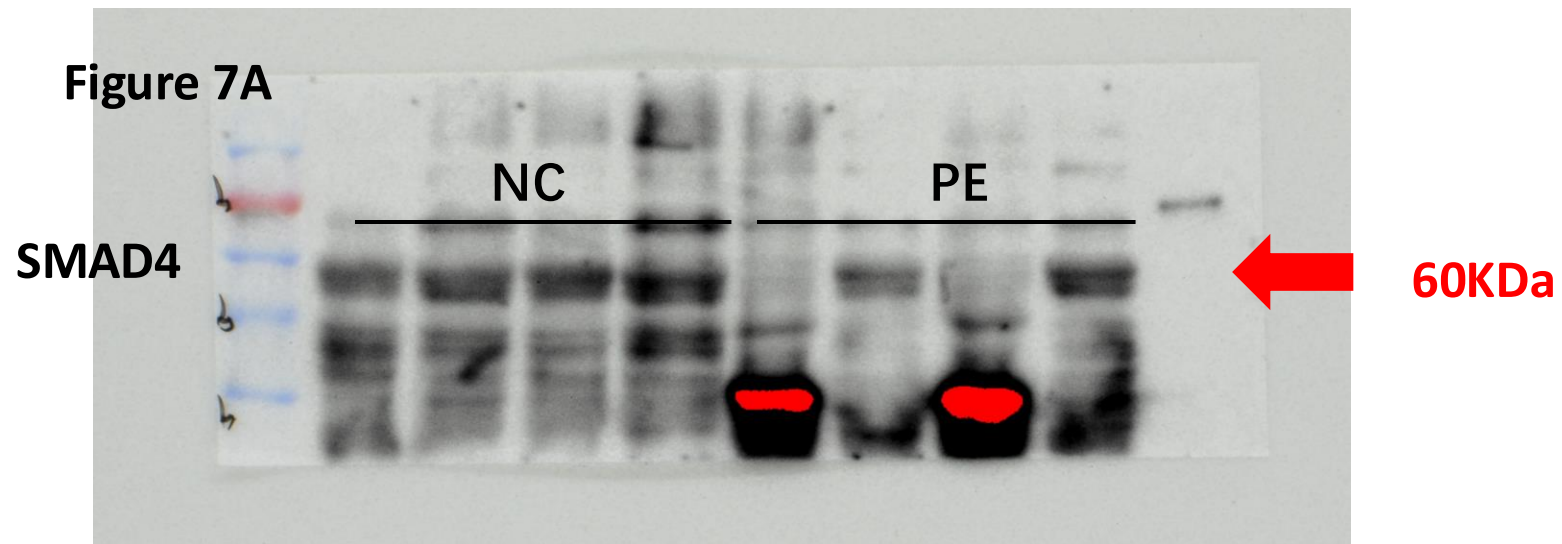

Supplement: Figure 7—source data 1. [file elife-101236-fig7-data1.zip › Figure 7A-SMAD4 source data 2.pdf]

Figure 7A

SMAD1/5

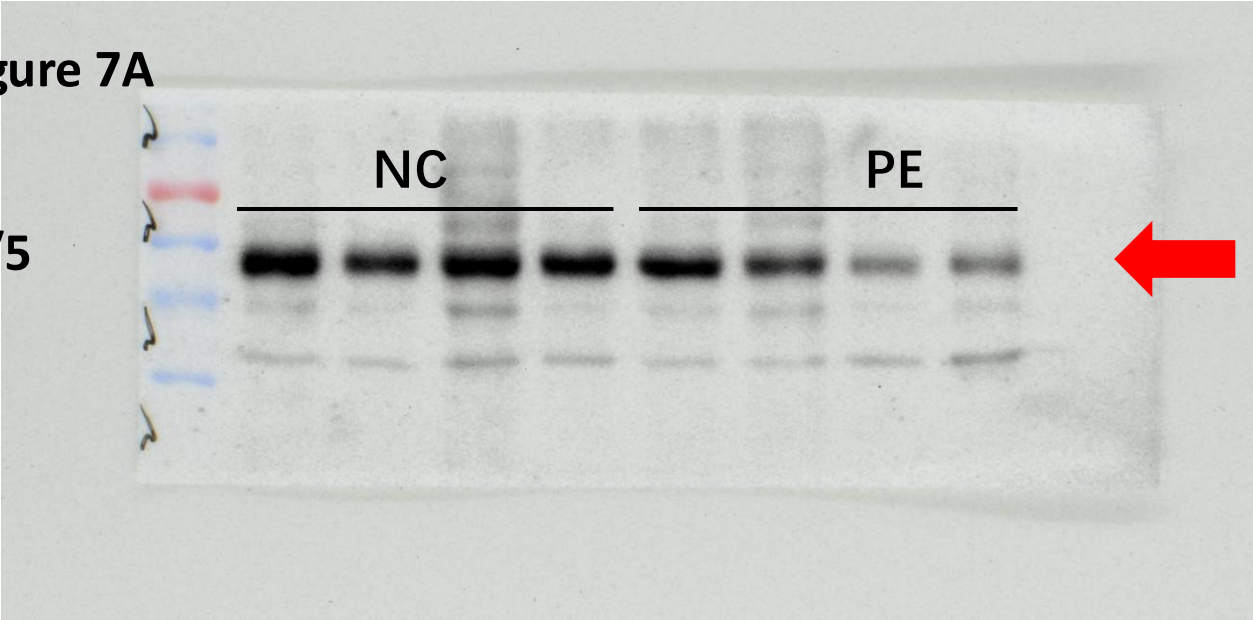

51KDa

Supplement: Figure 7—source data 1. [file elife-101236-fig7-data1.zip › Figure 7A-smad15 source data 1.pdf]

Figure 7A

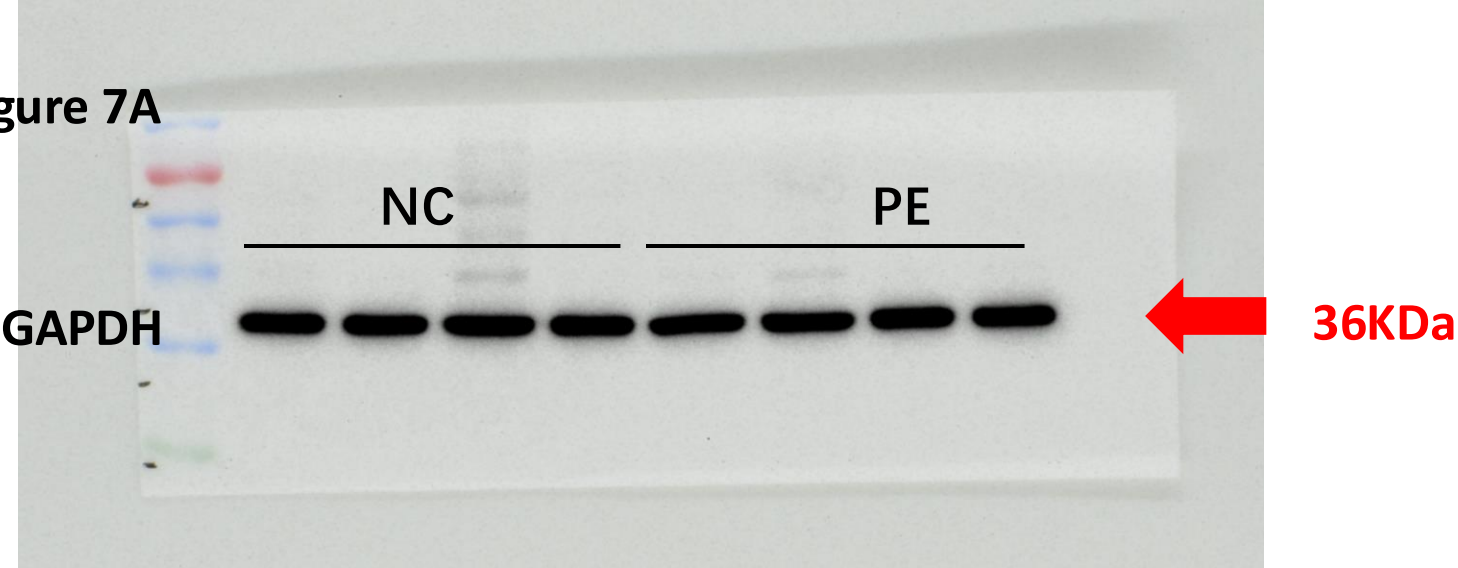

Supplement: Figure 7—source data 1. [file elife-101236-fig7-data1.zip › Figure 7A-smad15 source data 2.pdf]

Figure 7A

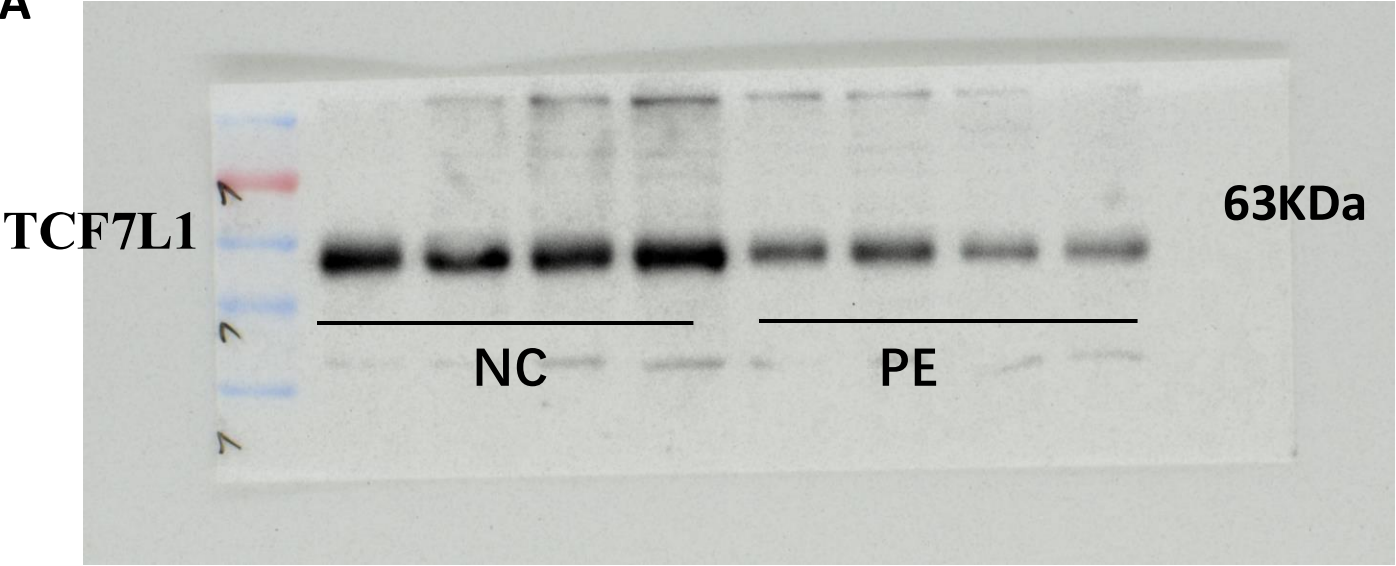

Supplement: Figure 7—source data 1. [file elife-101236-fig7-data1.zip › Figure 7A-TCF7L1 source data 1.pdf]

**Figure 7A**

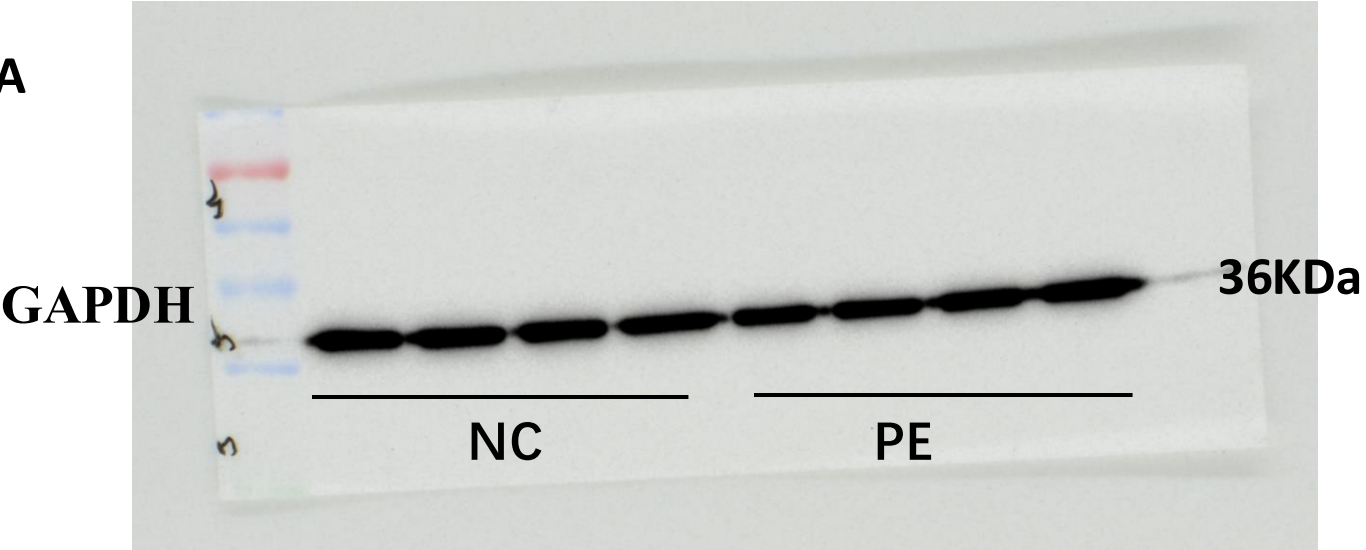

Supplement: Figure 7—source data 1. [file elife-101236-fig7-data1.zip › Figure 7A-TCF7L1 source data 2.pdf]

**Figure 7A**

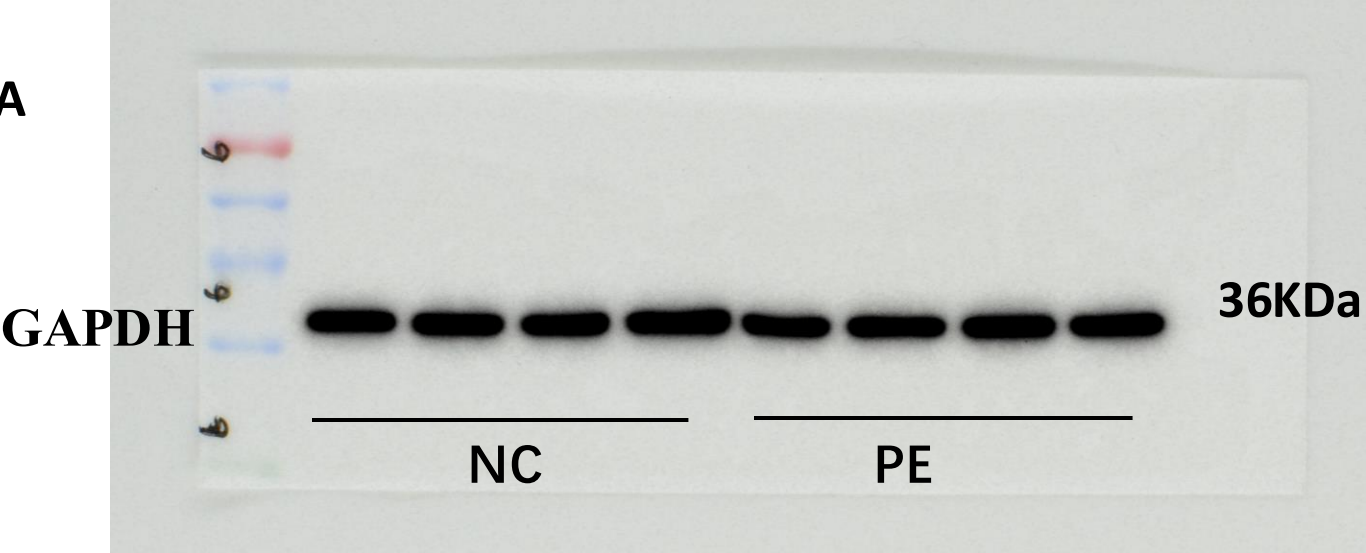

Supplement: Figure 7—source data 1. [file elife-101236-fig7-data1.zip › Figure 7A-TCF7L2 source data 1.pdf]

Figure 7A

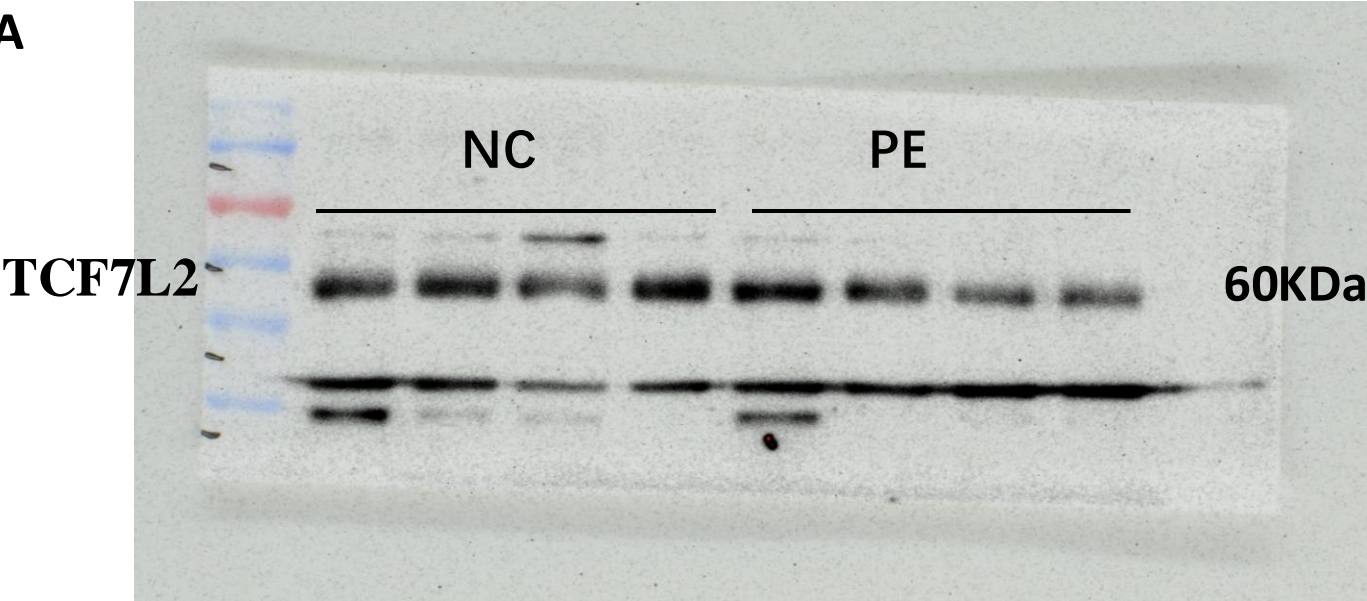

Supplement: Figure 7—source data 1. [file elife-101236-fig7-data1.zip › Figure 7A-TCF7L2 source data 2.pdf]

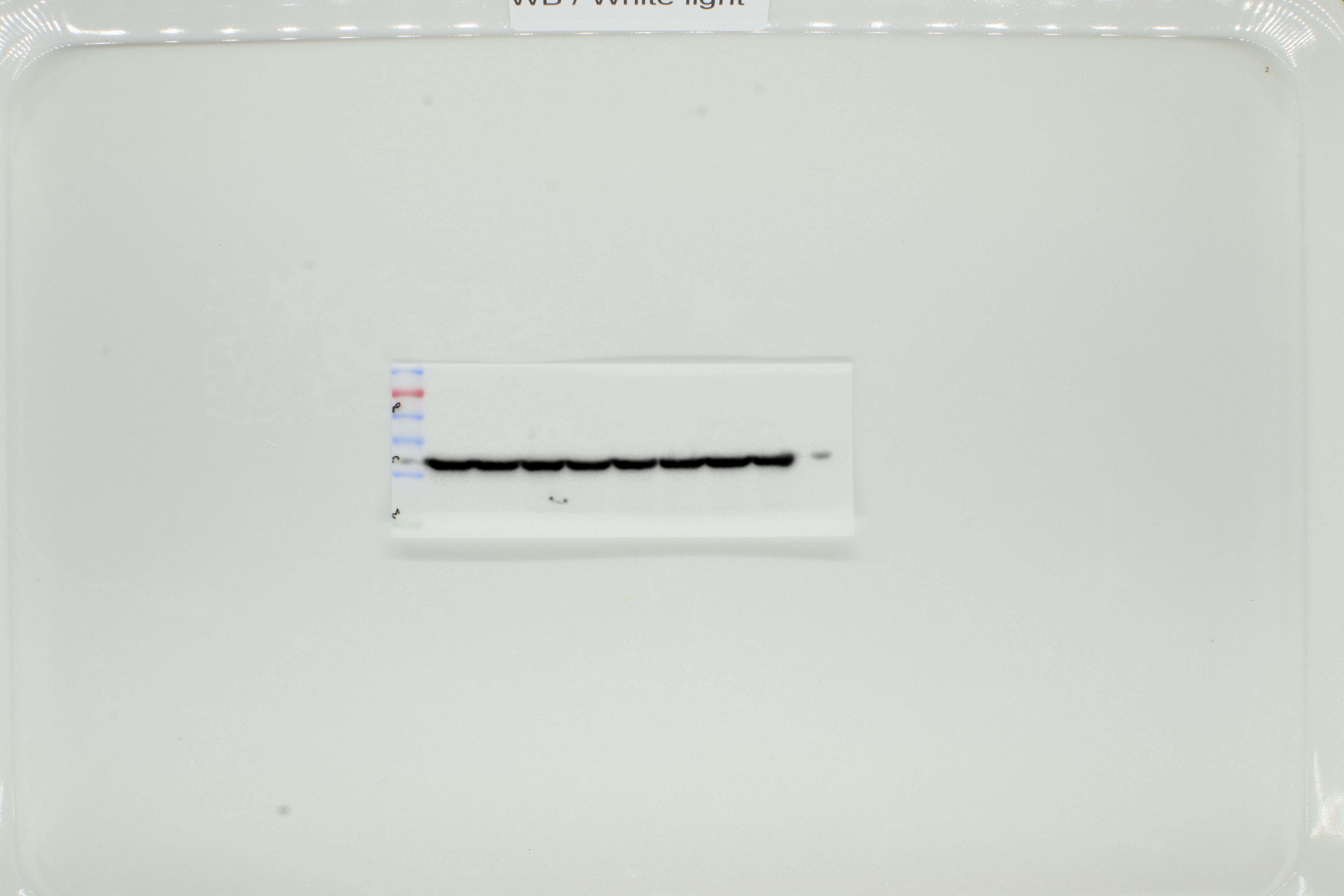

Supplement: Figure 7—source data 2. [file elife-101236-fig7-data2.zip › Figure 7A-ACVR2A source data 1.jpg]

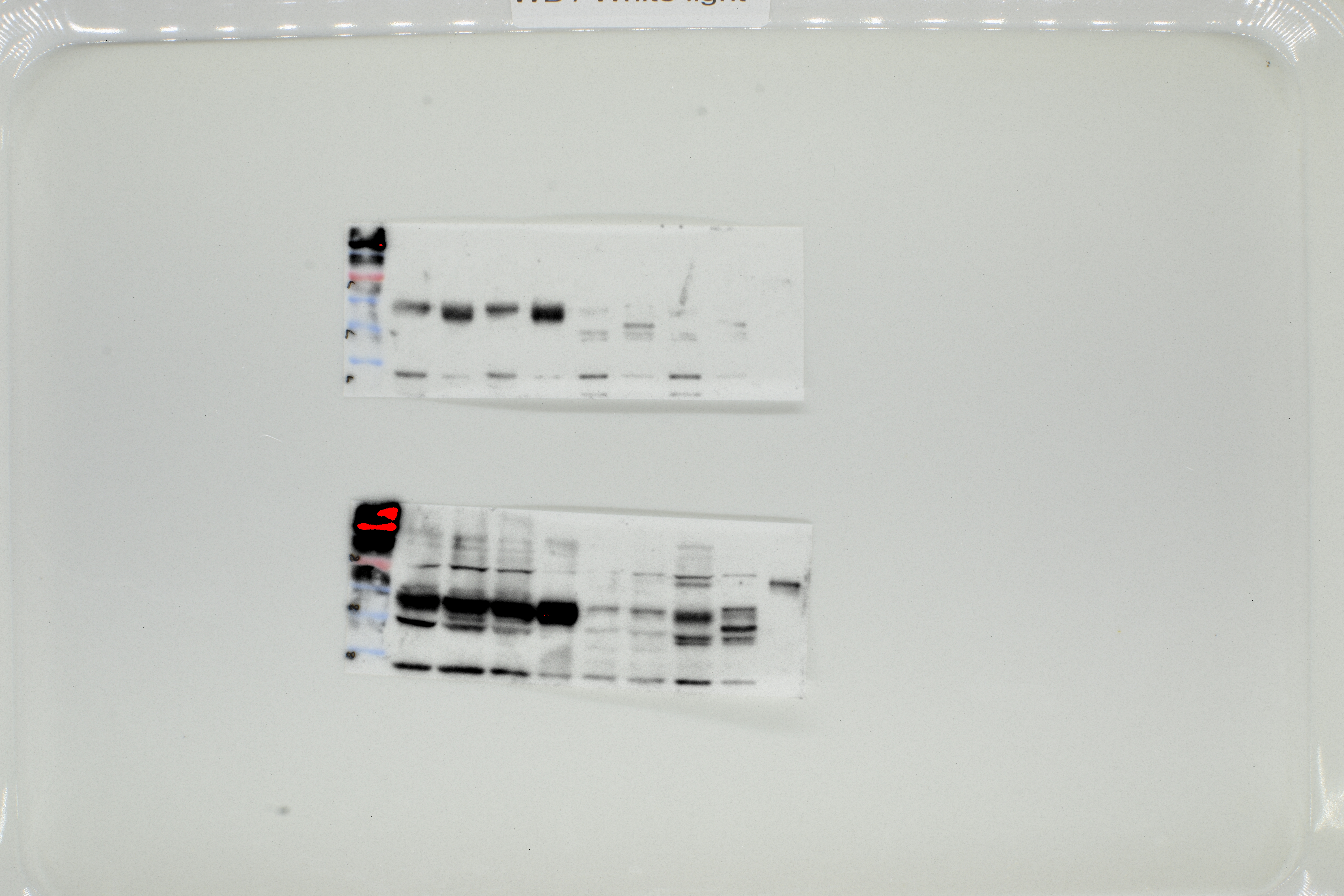

Supplement: Figure 7—source data 2. [file elife-101236-fig7-data2.zip › Figure 7A-ACVR2A source data 2.jpg]

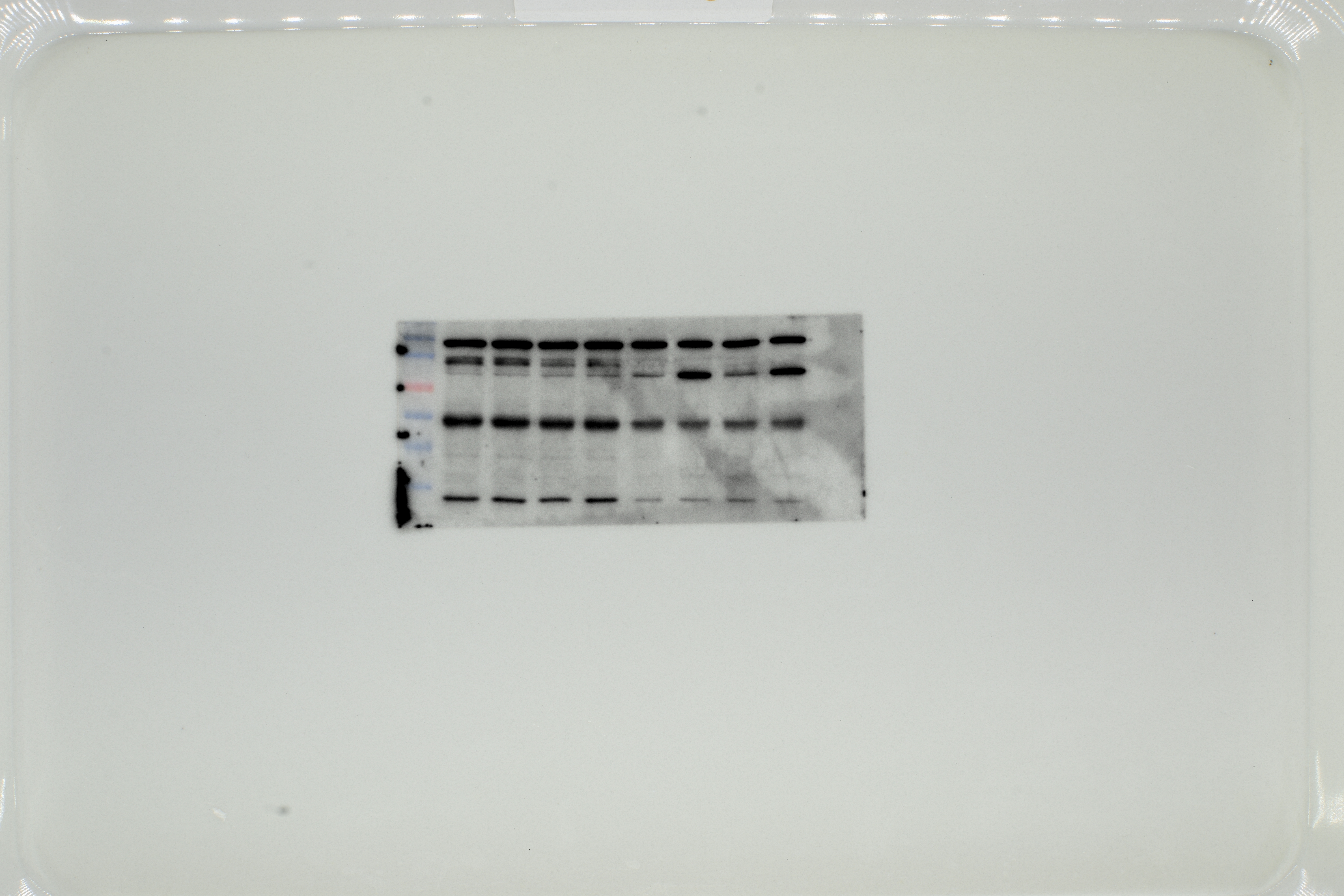

Supplement: Figure 7—source data 2. [file elife-101236-fig7-data2.zip › Figure 7A-pSMAD159 source data 1.jpg]

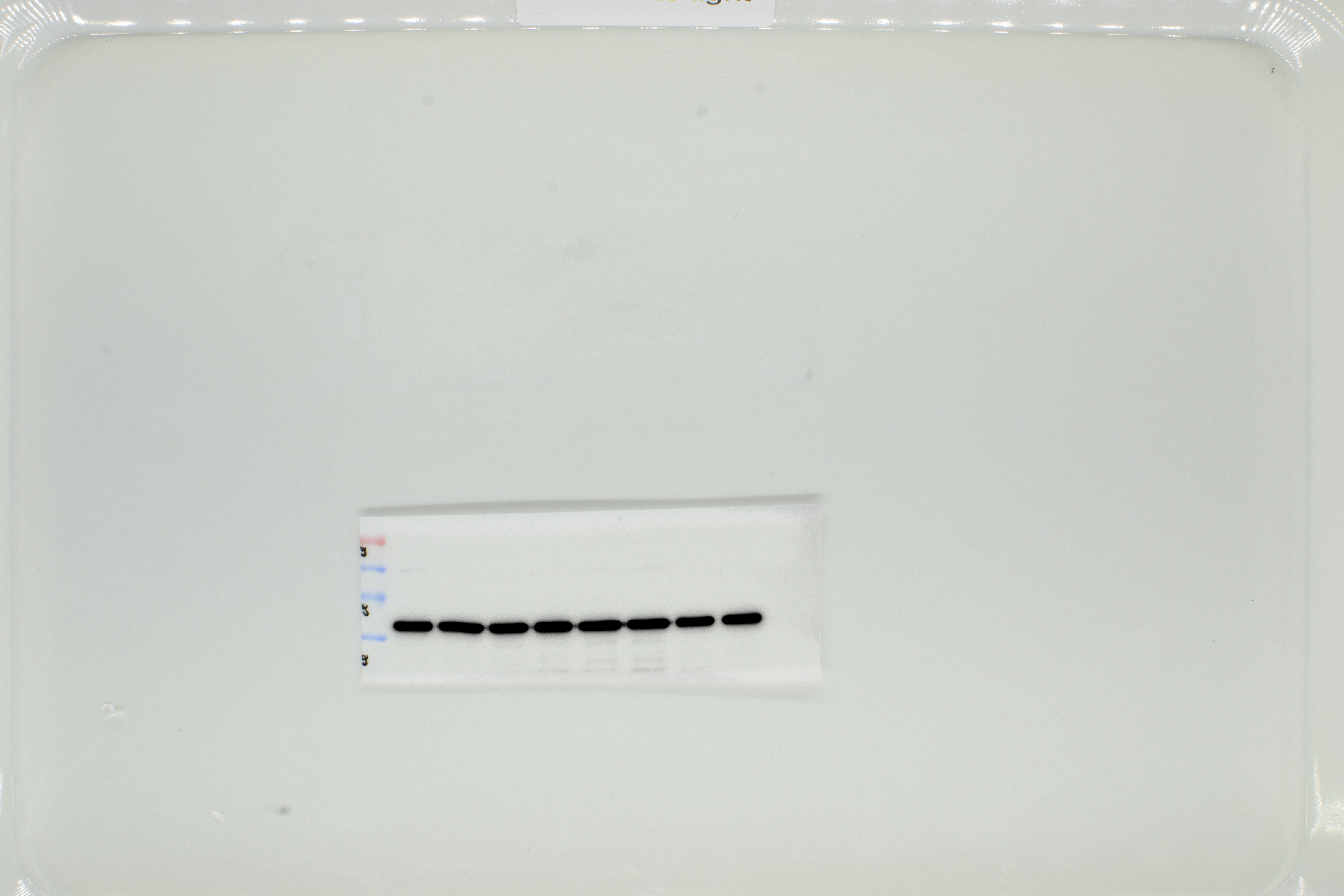

Supplement: Figure 7—source data 2. [file elife-101236-fig7-data2.zip › Figure 7A-pSMAD159 source data 2.jpg]

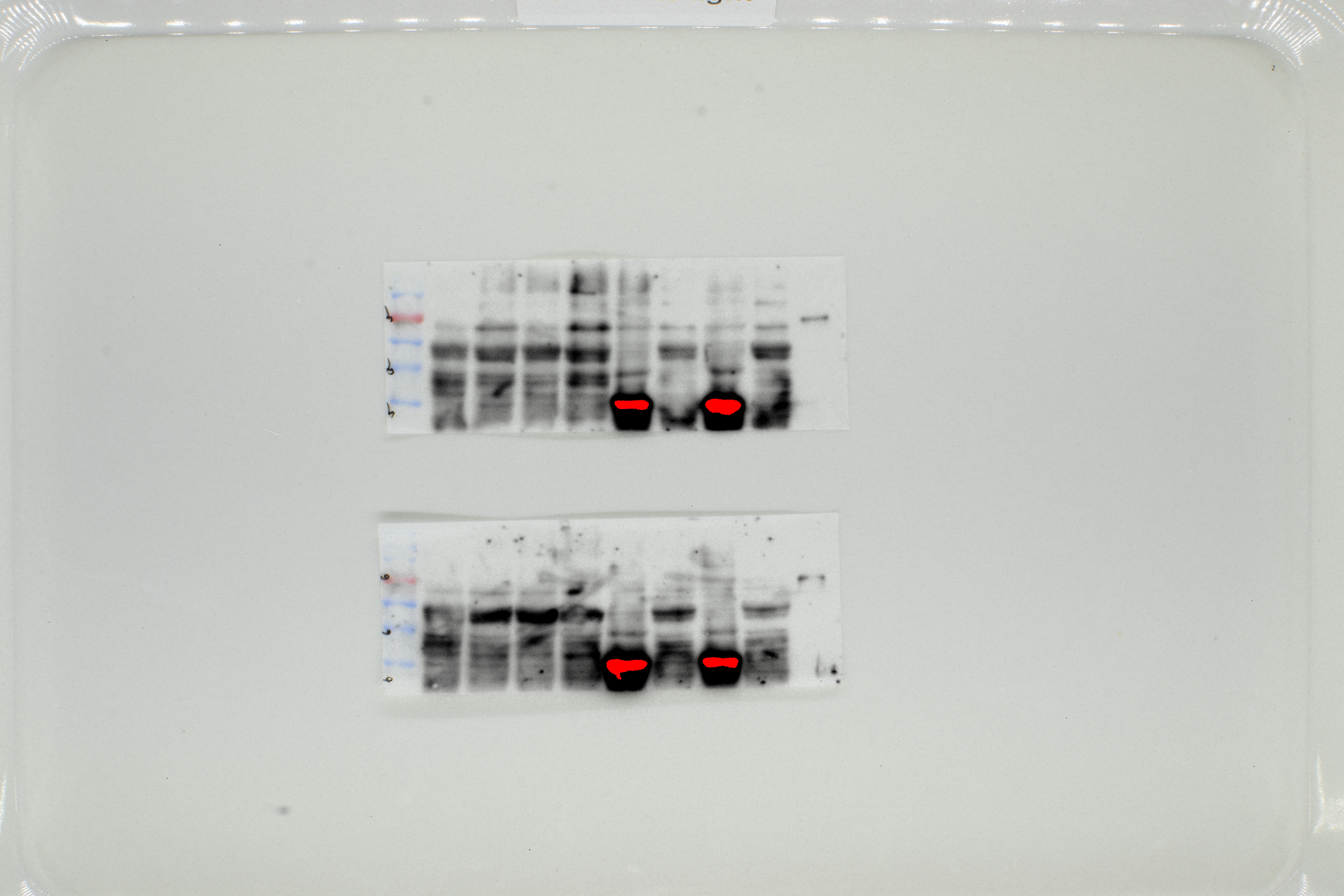

Supplement: Figure 7—source data 2. [file elife-101236-fig7-data2.zip › Figure 7A-SMAD4 source data 2.jpg]

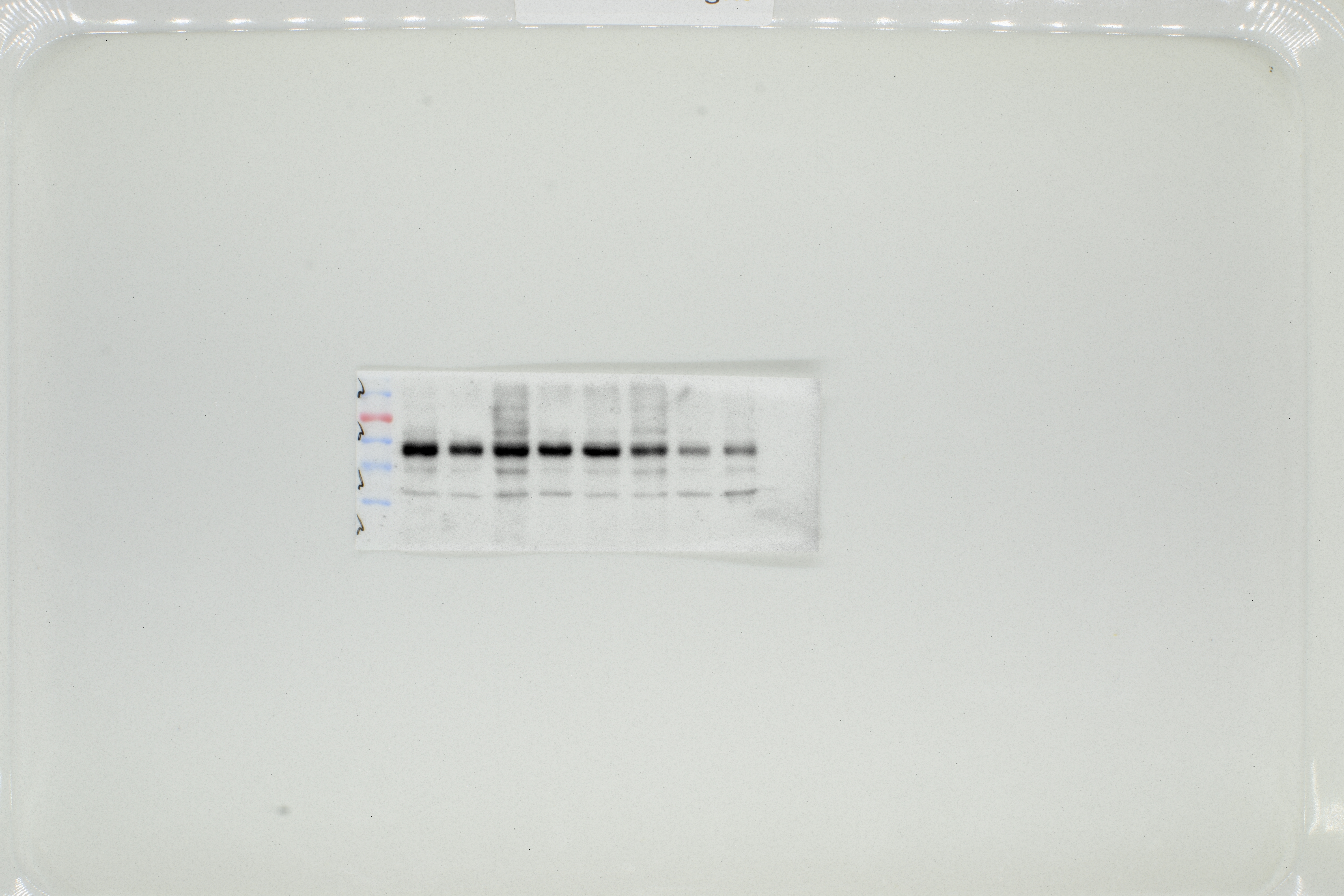

Supplement: Figure 7—source data 3. [file elife-101236-fig7-data3.zip › Figure 7A-smad15 source data 1.jpg]

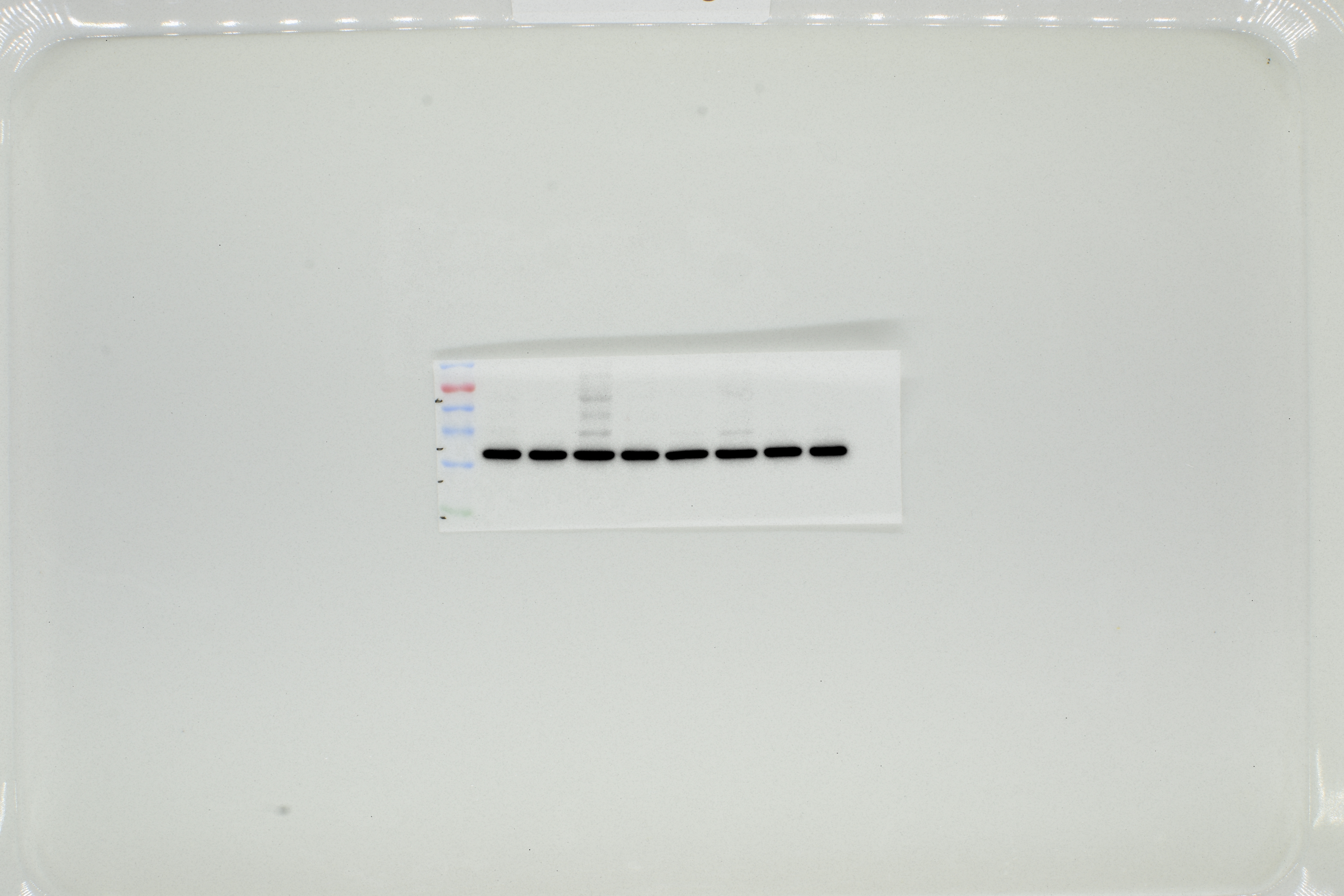

Supplement: Figure 7—source data 3. [file elife-101236-fig7-data3.zip › Figure 7A-smad15 source data 2.jpg]

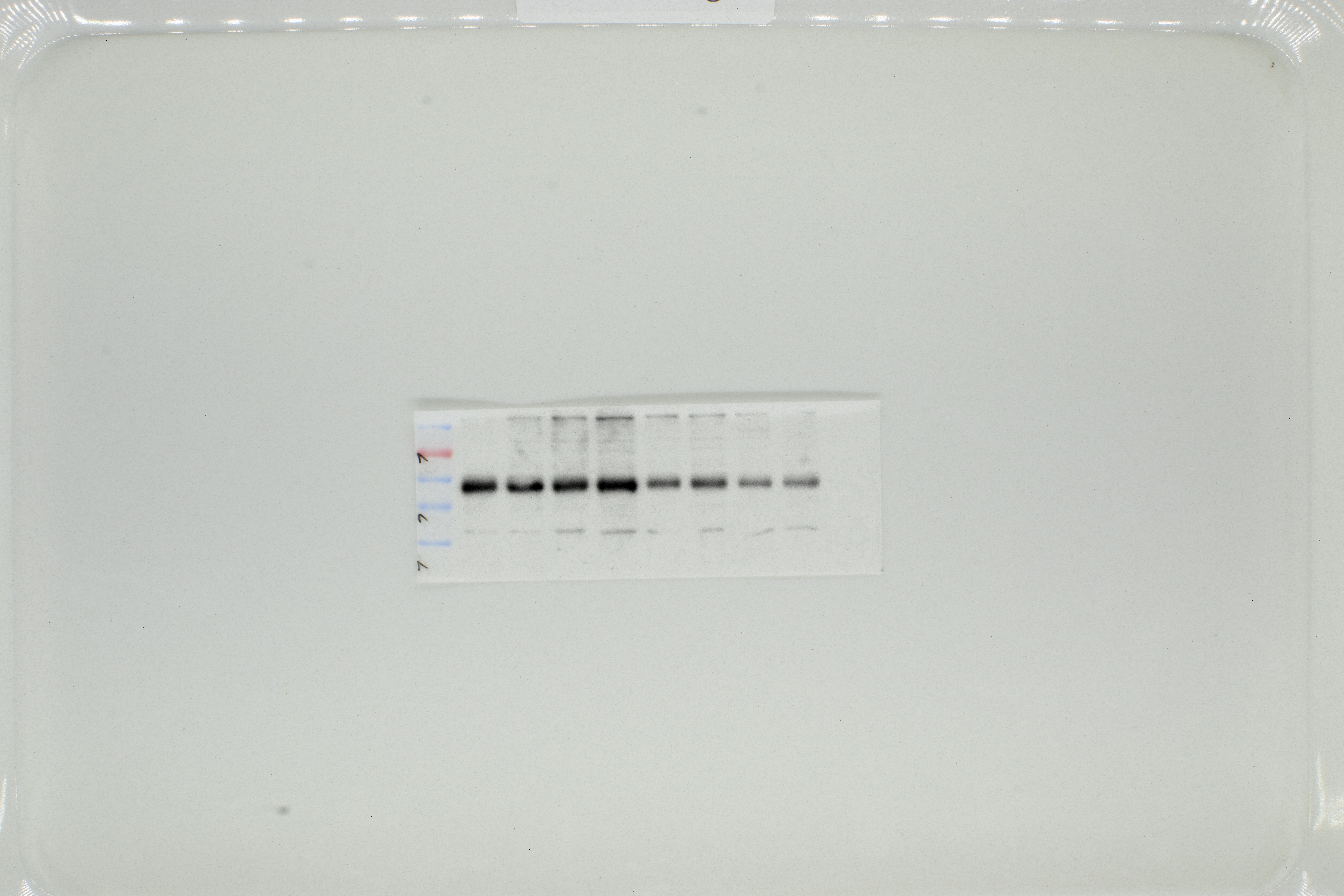

Supplement: Figure 7—source data 3. [file elife-101236-fig7-data3.zip › Figure 7A-TCF7L1 source data 1.jpg]

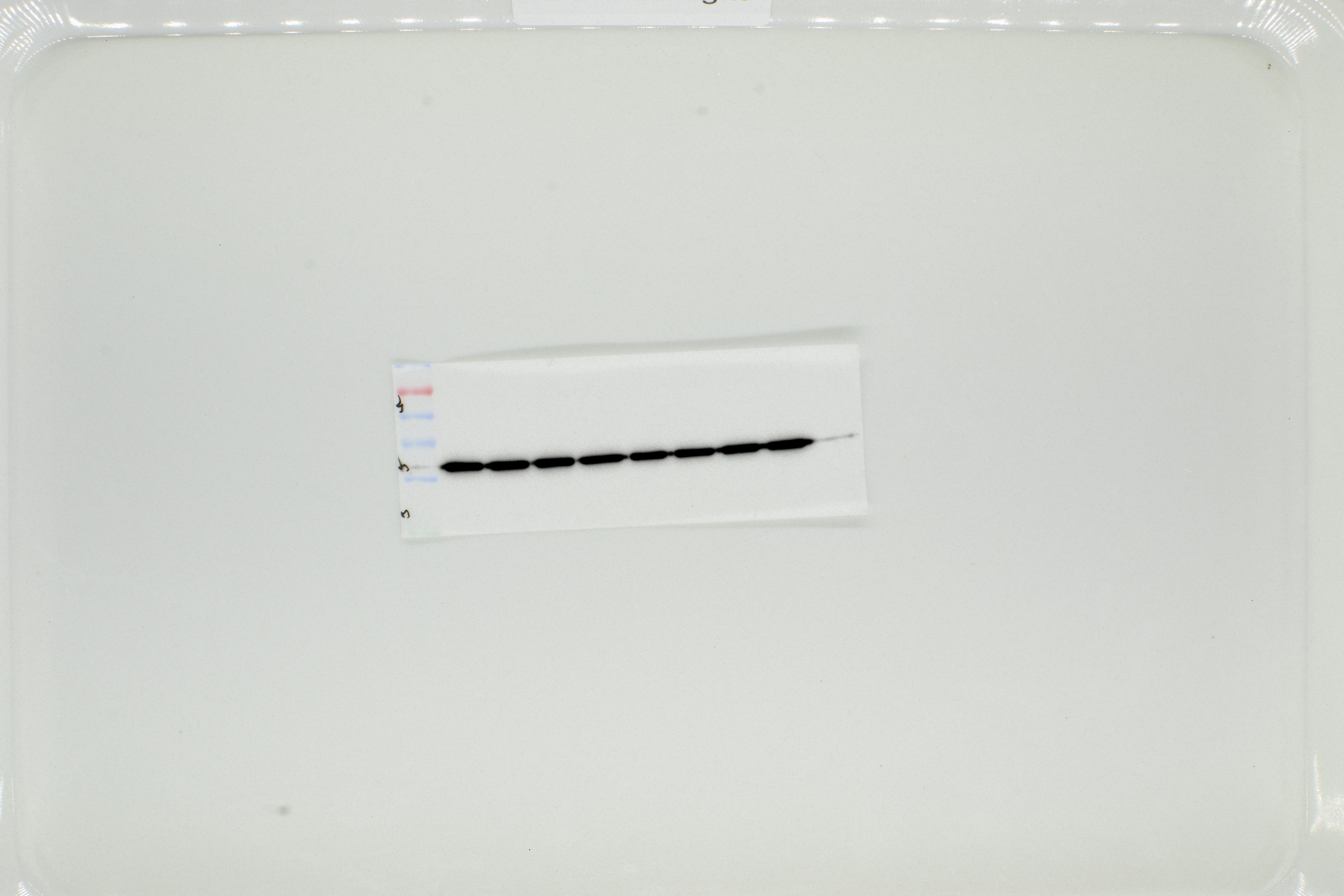

Supplement: Figure 7—source data 3. [file elife-101236-fig7-data3.zip › Figure 7A-TCF7L1 source data 2.jpg]

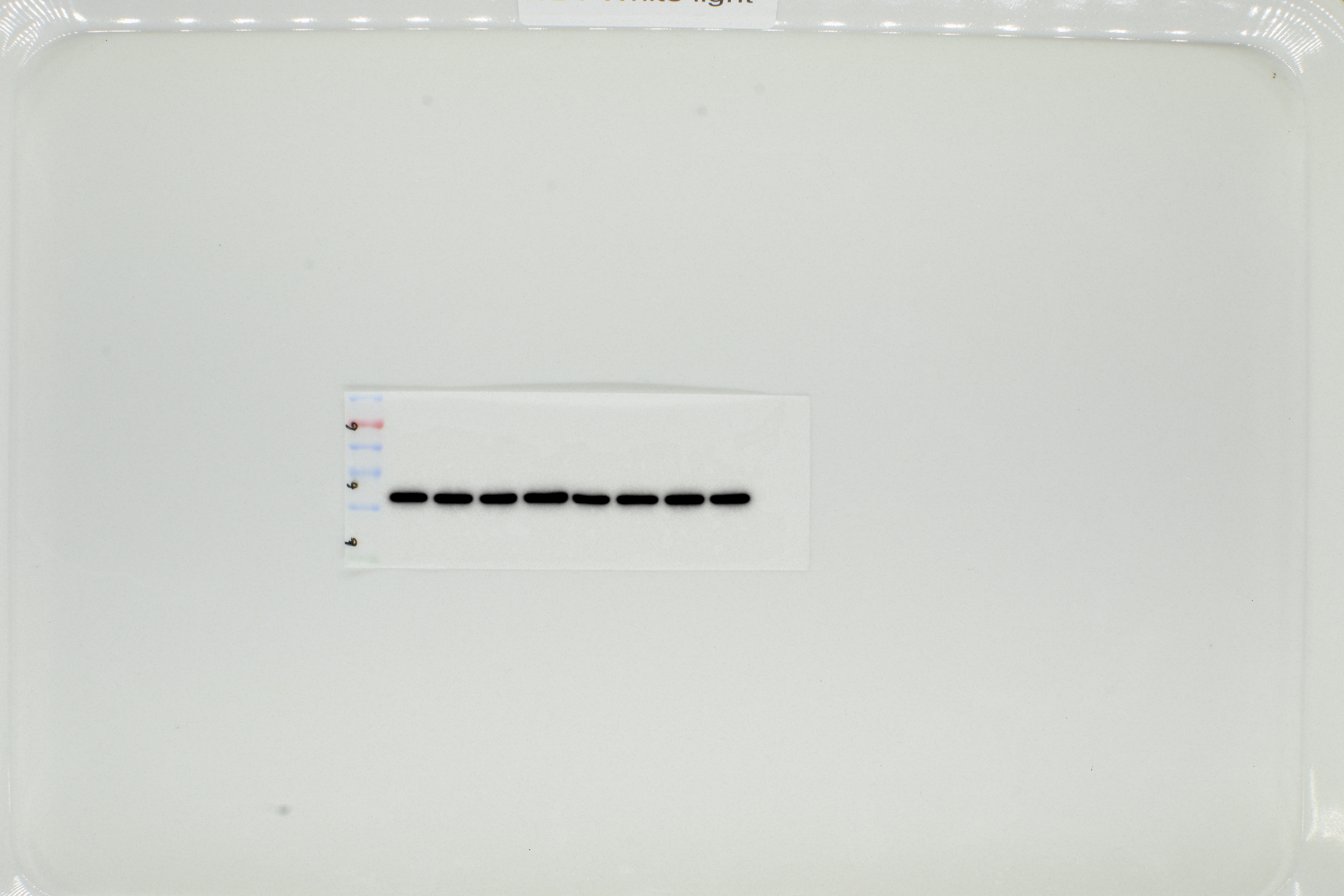

Supplement: Figure 7—source data 3. [file elife-101236-fig7-data3.zip › Figure 7A-TCF7L2 source data 1.jpg]

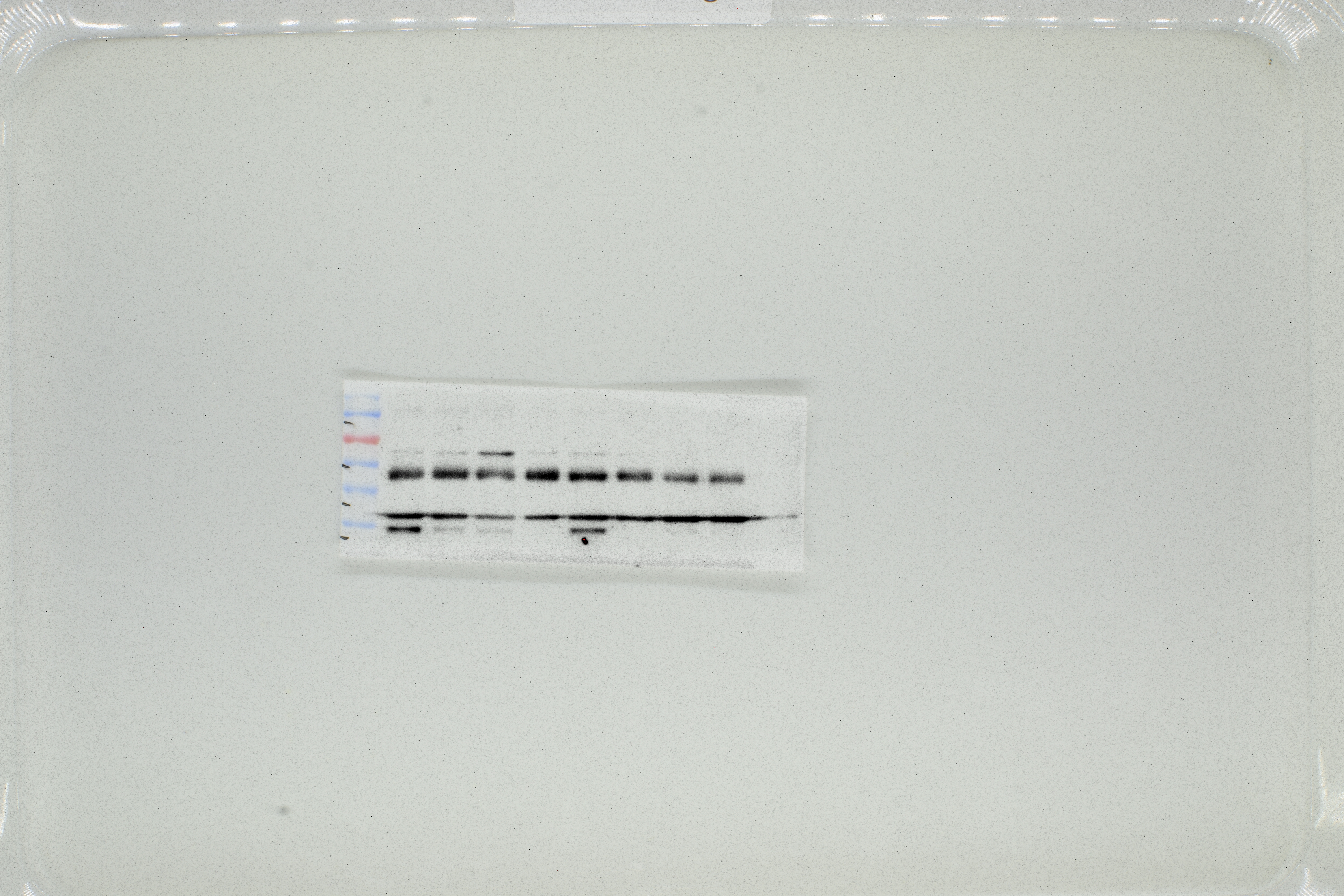

Supplement: Figure 7—source data 3. [file elife-101236-fig7-data3.zip › Figure 7A-TCF7L2 source data 2.jpg]
