# Supplementary material for: ACVR2A facilitates trophoblast cell invasion through TCF7/c-JUN pathway in pre-eclampsia progression
Source: eLife. 2025 May 30;14:RP101236. doi: 10.7554/eLife.101236 (PMC12124833; doi:10.7554/eLife.101236)
Supplement: Supplementary file 1. [file elife-101236-supp1.docx]

Table S1 Clinical characteristics of pre-eclamptic and normal control pregnancies.

| Variable | PE group (n=20, mean ± SD ) | NC group (n=20, mean ± SD) |
| --- | --- | --- |
| Age ^a^ | 33 ± 3 | 31.4 ± 2.44 |
| Predelivery BMI ^a^ (kg/m2) | 24.79± 3.22 | 23.34 ± 3.39 |
| Systolic blood pressure ^a^ (mmHg) | 149.50 ± 7.31 | 113.79 ± 9.97 |
| Diastolic blood pressure ^a^ (mmHg) | 97.65 ± 6.92 | 72.63 ± 5.88 |
| Gestational age ^a^ (week) | 36 (34, 38) | 38 (37, 39) |
| Neonatal Birth Weight (g) | 2738.75 ± 694.96 | 3221.50 ± 242.75 |

Continuous variables that conform to the normal distribution are represented by the mean sigma standard deviation, and those that do not conform to the normal distribution are represented by the median (P25, P75)

a The Mann-Whitney test was used for statistical analysis, median (IQR).

b The Chi-square test was used for statistical analysis.
