# Supplementary material for: ACVR2A facilitates trophoblast cell invasion through TCF7/c-JUN pathway in pre-eclampsia progression
Source: eLife. 2025 May 30;14:RP101236. doi: 10.7554/eLife.101236 (PMC12124833; doi:10.7554/eLife.101236)
Supplement: Supplementary file 2. [file elife-101236-supp2.docx]

Table S2. Sequences of the primers for RT-qPCR.

| Primer | | Sequence |
| --- | --- | --- |
| GAPDH-F | | 5’- AAAAGCATCACCCGGAGGAGAA -3’ |
| GAPDH-R | | 5’- GATAACCTGGCTTCTGCGTCGT -3’ |
| C-JUN-F | | 5’- GATAACCTGGCTTCTGCGTCGT -3’ |
| C-JUN-R | | 5’- TGCTGCGTTAGCATGAGTTGGC -3’ |
| TCF7-F | | 5’- CTGACCTCTCTGGCTTCTACTC -3’ |
| TCF7-R | | 5’- CAGAACCTAGCATCAAGGATGGG -3’ |
| TCF7L1-F | | 5’- TCAAGGACACGAGGTCACCATC -3’ |
| TCF7L1-R | | 5’- GGAGAAGTGGTCATTGCTGTAGG -3’ |
| Wnt3-F | | 5’- GCGTGTTAGTGTCCAGGGAGTT -3’ |
| Wnt3-R | 5’- TGAGGTGCATGTGGTCCAGGAT -3’ | |
| Wnt4-F | 5’- ATGAACCTCCACAACAATGAG -3’ | |
| Wnt4-R | 5’- ACCATCAAACTTCTCCTTCAG -3’ | |
| SMAD4-F | 5’- CTACCAGCACTGCCAACTTTCC -3’ | |
| SMAD4-R | 5’- CCTGATGCTATCTGCAACAGTCC -3’ | |
| CCND1-F | 5’- TCTACACCGACAACTCCATCCG -3’ | |
| CCND1-R | 5’- TCTGGCATTTTGGAGAGGAAGTG -3’ | |
| ACVR2A-F | 5’- GCCAGCATCCATCTCTTGAAGAC -3’ | |
| ACVR2A-R | 5’- GATAACCTGGCTTCTGCGTCGT -3’ | |
