# Supplementary material for: ACVR2A facilitates trophoblast cell invasion through TCF7/c-JUN pathway in pre-eclampsia progression
Source: eLife. 2025 May 30;14:RP101236. doi: 10.7554/eLife.101236 (PMC12124833; doi:10.7554/eLife.101236)
Supplement: Supplementary file 3. [file elife-101236-supp3.docx]

Table S3. Sequences of the genotyping primers.

| Primer | Sequence |
| --- | --- |
| ACVR2A-F | 5’- ACTGATACTGCTCAGTGGTGAC -3’ |
| ACVR2A-R | 5’- CCCTTGTTCATAACCCAGGTC -3’ |
