# Supplementary material for: ACVR2A facilitates trophoblast cell invasion through TCF7/c-JUN pathway in pre-eclampsia progression
Source: eLife. 2025 May 30;14:RP101236. doi: 10.7554/eLife.101236 (PMC12124833; doi:10.7554/eLife.101236)
Supplement: Supplementary file 4. [file elife-101236-supp4.docx]

Table S4. Details of immunohistochemical antibodies

| Antibody | corporation | Cat | concentration |
| --- | --- | --- | --- |
| ACVR2A | Thermo | PA5-95374 | 1:300 |
| HLA-G | proteintech | 66447-1-IG | 1:100 |
| Wnt3 | bioss | bs-1700R | 1:100 |
| Wnt4 | bioss | bs-20786R | 1:100 |
| c-JUN | Servicebio | GB11071 | 1:1000 |
| CCND1 | Servicebio | GB111372 | 1:600 |
| TCF7L1 | bioss | bs-12891R | 1:100 |
| TCF7L2 | bioss | bs-1280R | 1:100 |
| SMAD4 | bioss | bsm-52225R | 1:100 |
| SMAD1/5 | bioss | bs-2973R | 1:100 |
| pSMAD1/5/9 | CellSignalingTechnology | 13820T | 1:100 |
